# Onvansertib treatment overcomes olaparib resistance in high-grade ovarian carcinomas

**DOI:** 10.1038/s41419-024-06894-1

**Published:** 2024-07-22

**Authors:** Michela Chiappa, Alessandra Decio, Luca Guarrera, Ilaria Mengoli, Anju Karki, Divora Yemane, Carmen Ghilardi, Eugenio Scanziani, Simone Canesi, Maria C. Barbera, Ilaria Craparotta, Marco Bolis, Robert Fruscio, Chiara Grasselli, Tommaso Ceruti, Massimo Zucchetti, Jesse C. Patterson, Robin A. Lu, Micheal B. Yaffe, Maya Ridinger, Giovanna Damia, Federica Guffanti

**Affiliations:** 1 Laboratory of Preclinical Gynecological Oncology, Experimental Oncology Department, Istituto di Ricerche Farmacologiche Mario Negri IRCCS, Milan, Italy; 2 Laboratory of Cancer Metastasis Therapeutics, Experimental Oncology Department, Istituto di Ricerche Farmacologiche Mario Negri IRCCS, Milan, Italy; 3 Computational Oncology Unit, Experimental Oncology Department, Istituto di Ricerche Farmacologiche Mario Negri IRCCS, Milan, Italy; 4 R&D Department, Cardiff Oncology, San Diego, CA USA; 5 Department of Veterinary Medicine and Animal Sciences (DIVAS), University of Milan, Lodi Campus, Italy; 6 Mouse and Animal Pathology Lab (MAPLab), UniMi Foundation, Milan, Italy; 7 Clinic of Obstetrics and Gynecology, Department of Medicine and Surgery, San Gerardo Hospital, University of Milan Bicocca, Monza, Italy; 8 Immuno-Pharmacology Unit, Department of Oncology, Mario Negri Institute for Pharmacological Research (IRCCS), Milan, Italy; 9 Laboratory of Laboratory of Cancer Pharmacology, Experimental Department of Oncology, Istituto di Ricerche Farmacologiche Mario Negri IRCCS, Milano, Italy; 10 Center for Precision Cancer Medicine, David H. Koch Institute for Integrative Cancer Research, Departments of Biology and Biological Engineering, Massachusetts Institute of Technology, Cambridge, MA USA

**Keywords:** Cancer therapy, Cancer models

## Abstract

Occurrence of resistance to olaparib, a poly(ADP-ribose) polymerase (PARP) inhibitor (PARPi) approved in ovarian carcinoma, has already been shown in clinical settings. Identifying combination treatments to sensitize tumor cells and/or overcome resistance to olaparib is critical. Polo-like kinase 1 (PLK1), a master regulator of mitosis, is also involved in the DNA damage response promoting homologous recombination (HR)-mediated DNA repair and in the recovery from the G2/M checkpoint. We hypothesized that PLK1 inhibition could sensitize tumor cells to PARP inhibition. Onvansertib, a highly selective PLK1 inhibitor, and olaparib were tested in vitro and in vivo in *BRCA1* mutated and wild-type (wt) ovarian cancer models, including patient-derived xenografts (PDXs) resistant to olaparib. The combination of onvansertib and olaparib was additive or synergic in different ovarian cancer cell lines, causing a G2/M block of the cell cycle, DNA damage, and apoptosis, much more pronounced in cells treated with the two drugs as compared to controls and single agents treated cells. The combined treatment was well tolerated in vivo and resulted in tumor growth inhibition and a statistically increased survival in olaparib-resistant-*BRCA1* mutated models. The combination was also active, although to a lesser extent, in *BRCA1* wt PDXs. Pharmacodynamic analyses showed an increase in mitotic, apoptotic, and DNA damage markers in tumor samples derived from mice treated with the combination versus vehicle. We could demonstrate that in vitro onvansertib inhibited both HR and non-homologous end-joining repair pathways and in vivo induced a decrease in the number of RAD51 foci-positive tumor cells, supporting its ability to induce HR deficiency and favoring the activity of olaparib. Considering that the combination was well tolerated, these data support and foster the clinical evaluation of onvansertib with PARPis in ovarian cancer, particularly in the PARPis-resistant setting.

## Introduction

Inhibitors of poly(ADP-ribose) polymerase (PARPis) represent the first clinically approved anticancer agents targeting the DNA damage response (DDR) pathway. They have been approved as monotherapy and in combination or maintenance settings in different tumor types, including high-grade ovarian carcinoma (HGOC) [1]. The efficacy of PARPis was first underlined in cells with functional inactivation of *BRCA1* and *BRCA2* genes, and this strong preclinical evidence prompted their clinical development in tumors with *BRCA1/BRCA2* mutations [2]. It was later demonstrated that PARPis were very effective in tumors displaying deficiency in homologous recombination (HR) repair beyond *BRCA1* and *BRCA2* loss of function. In addition, evidence from randomized clinical trials supports their efficacy also in tumors with intact HR repair, although to a lesser extent [3].

PARP1 is a key DNA repair protein, able to detect and bind DNA single strand breaks with the subsequent PARylation (including auto-PARylation) of proteins that promote chromatin remodeling and facilitate the recruitment of DNA repair proteins [4]. PARPis’ efficacy on HR-deficient cells relies on their ability to trap PARP1 onto DNA, generating DNA double-strand breaks (DSBs) during replication. In an HR-deficient background, these DSBs can only be repaired by alternative pathways, including the error-prone non-homologous end joining (NHEJ) pathway, leading to the accumulation of DNA damage, genomic instability, and cell death [5]. Recently, the involvement of microhomology-mediated end joining (MMEJ) repair and the formation of gaps during repair have been demonstrated to be important for PARPi-induced cytotoxicity [6].

With the widespread use of PARPis in different tumor types, clinical resistance is emerging, and it will represent a major clinical challenge in the near future. Multiple mechanisms of PARPi resistance have been highlighted in preclinical models, and some of them confirmed in the clinical setting [4, 7]. Possible mechanisms of resistance involve restoration of the HR repair pathway through either *BRCA*-dependent (*e.g*. reversion mutations and epigenetic upregulation of *BRCA1*) or *BRCA*-independent mechanisms (e.g., loss of p53-binding protein 1 [*53BP1*] or *REV7*; or reversion mutations of non-*BRCA* HR pathway genes, like *RAD51C* or partner and co-localizer of *BRCA2*-*PALB2*). Additionally, HR-independent mechanisms, including mutations in *PARP1*, loss of the dePARylation enzyme poly(ADP-ribose) glycohydrolase, restoration of replicative fork stability, and upregulation of secondary signaling pathways have been recently proposed [7].

Polo-like kinase 1 (PLK1) is a serine-threonine kinase and a master regulator of mitosis, where its functions are well understood [8, 9]. Additional experimental evidence suggests it functions outside of mitosis as modulator of the DDR and G2/M checkpoint resolution after DNA damage [10, 11]. Adriamycin treatment has been reported to inhibit PLK1 activity and likely prevent the activation of the G2/M checkpoint [12]. RAD51 S14 phosphorylation by PLK1 has been observed 20–40 minutes after DNA damage and has been shown to promote HR [13, 14]; this event facilitates CK2-mediated RAD51 phosphorylation at T13. Several experimental observations support both the recruitment of PLK1 to stripes of UV-damaged DNA [15] and DNA DSBs [16, 17], and that PLK1-dependent S723 phosphorylation of the C-terminal binding protein interacting protein (CtIP) facilitates error-prone MMEJ and inactivation of the G2*/*M checkpoint [18]. It has recently been shown that not only is PLK1 recruited to DNA damage sites in a PARP1-dependent manner, but also that CHK1 phosphorylates PLK1 at S137, and then subsequently at T210 to promote its full enzymatic activity toward RAD51 thus enhancing HR repair [16]. Phosphorylation of Mre11 at S649/S688 by PLK1 inhibits the loading of the MRN complex to damaged DNA, causing both premature DNA damage checkpoint termination and inhibition of DNA repair; tumors expressing phospho-mimetic Mre11 are more sensitive to the PARPi olaparib [19]. Very recently, PLK1 has been reported to phosphorylate polymerase theta (Polθ), with its subsequent recruitment to mitotic DSBs, where Polθ can mediate the joining of the DNA broken ends [20].

Onvansertib is an orally available, highly selective PLK1 inhibitor undergoing clinical investigation [21, 22]. It has been described to be active in combination with ionizing radiation, cytarabine, irinotecan, paclitaxel, and abiraterone in different preclinical models, with very interesting additive or synergistic activities reported [23–29].

Given the roles of PLK1 in the DDR pathway, the aim of the present work was to explore the effect of onvansertib alone or in combination with olaparib in olaparib-resistant ovarian cancer preclinical models. Data reported suggest that the combination is very active in vivo resulting in tumor growth inhibition and prolongation of median survival time with no observed cumulative toxicity. Greater antitumor activity was observed in *BRCA1* mutated compared to *BRCA* wild-type (wt) patient-derived xenograft (PDX) models. Mechanistic data suggest that the combination causes an increase in DNA damage in tumor cells leading to cell death. These data warrant the clinical exploitation of this regimen in ovarian cancer patients with olaparib-resistant tumors.

## Results

### Cytotoxic activity of onvansertib and olaparib in multiple ovarian cancer cell lines

**Fig. 1:**
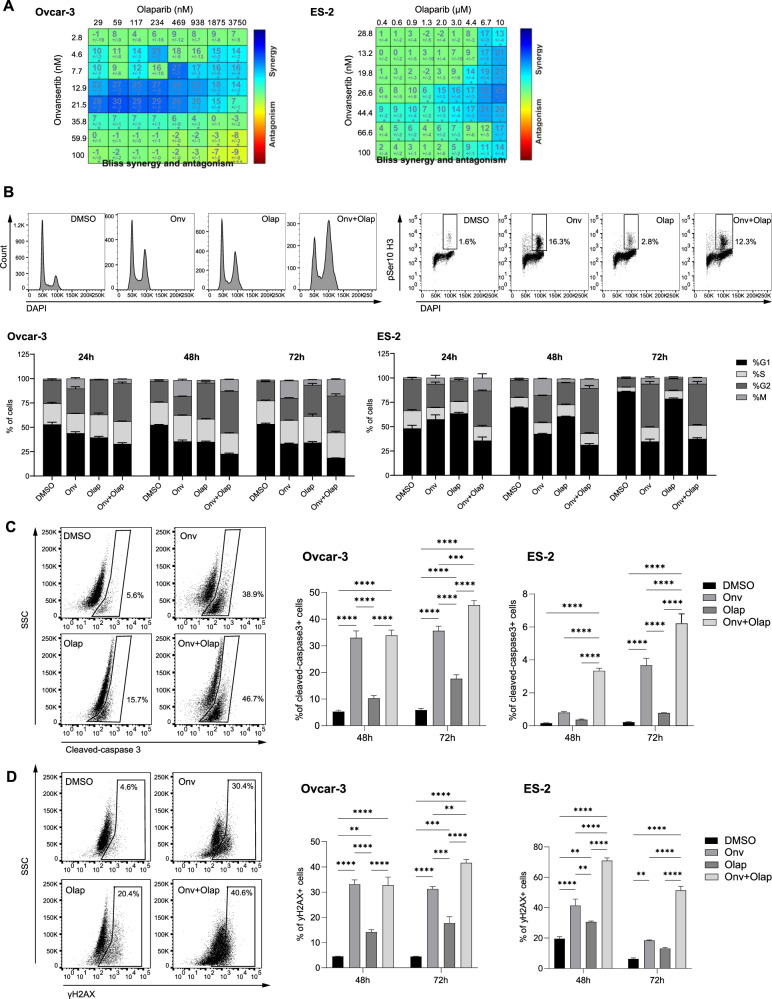
Cytotoxicity, cell cycle perturbation, apoptosis induction, and DNA damage after olaparib and onvansertib single treatment and their combination in Ovcar-3 and ES-2 cell lines. **A** Ovcar-3 (left) and ES-2 (right) cells were treated for 6 days with increasing dose of onvansertib and olaparib. Drug synergy is indicated by blue squares within the Bliss Synergy Heatmap. **B**–**D** Ovcar-3 and ES-2 cells were treated with DMSO, olaparib (Ola), onvansertib (Onv), or the combination (Onv+Ola). Doses used are 30 or 50 nM Onv and 2.5 or 5 μM Ola for Ovcar-3 and ES-2 cells, respectively. At the indicated timepoints, cells were fixed, permeabilized, and stained with antibodies against phospho-histone 3 (pSer10, **B**), cleaved-caspase 3 (**C**), or γH2AX (**D**); the DNA was stained with DAPI. Cells were analyzed by flow cytometry. Representative histograms show DAPI-stained DNA content. Representative scatter plots show the percentage of cells positive for the indicated stain. Bar graphs show the quantification of the % of cells in different cell cycle phases (**B**) and the % of positive cells for each marker (**C**, **D**). Data are presented as mean ± SEM of 3 independent experiments. Two-way ANOVA was used to test statistical differences. **p < 0.01, ***p < 0.001, ****p < 0.0001.

The effect of onvansertib and olaparib on cell viability was tested in two human ovarian cancer cell lines: Ovcar-3 (*TP53* loss, *CCNE1* amplified and *BRCA* wt) and ES-2 (*TP53* loss, *BRCA* wt, *PALB2* mutated); in parental p53 deleted ID8 murine syngeneic cell lines (F3-*BRCA1* wt- and F3-*BRCA1*−/−) and their corresponding olaparib-resistant cell lines (F3-OlaR and F3-*BRCA1*−/−OlaR), obtained in our laboratory [30]. As shown in Fig. 1A and Supplementary Fig. S1, the combination was found to be synergistic or additive in human and murine cell lines. In contrast to what has been reported [31], F3-*BRCA1* wt and F3-*BRCA1*−/− cells showed similar sensitivity to onvansertib. In both, F3-*BRCA1* wt and *BRCA1*−/− cells, the combination was also active in olaparib-resistant cells (Supplementary Fig. S1). Moreover, onvansertib treatment synergized with two other PARPis, suggesting that this is not unique to olaparib (Supplementary Fig. S2).

We then examined the effect of onvansertib and olaparib on the cell cycle at 24 h, 48 h, and 72 h. For this, Ovcar-3 and ES-2 cells were treated with single agents (with drug doses in the range of IC_30_-IC_50_) or the combination, and cell cycle distribution was analyzed by flow cytometry using DAPI (4’,6-diamidino-2-phenylindole), and antibodies against phospho- S10 histone H3 (pSer10 H3), a marker of mitotic cells (Fig. 1B). Onvansertib resulted in a significant increase in G2/M cells at 24 h, 48 h and 72 h in Ovcar-3 cells and at 48 h and 72 h in ES-2 cells, consistent with its roles in mitotic entry and progression [32, 33]. Olaparib induced G2 arrest in Ovcar-3 cells at all timepoints, while marginal cell cycle perturbations were observed in ES-2 cells. A significant increase in G2/M cells was observed in Ovcar-3 and ES-2 cells treated with the combination compared to control and single agent-treated cells, with >50% of cells in G2/M at 48 h in both cell lines. The G2/M block was also corroborated by a high amount of cyclin B1 in Ovcar-3 cells treated with the combination (Supplementary Fig. S3A).

Cell apoptosis was assessed by quantifying cleaved-caspase-3 positive cells (Fig. 1C) via Fluorescence-activated Cell Sorting (FACS). Increased apoptosis was observed in the combination group compared to DMSO- and single agent-treated cells. Western blot analysis of cleaved-caspase-3 and cleaved-PARP confirmed increased apoptosis in cells treated with the combination (Supplementary Fig. S3B). Finally, we assessed the changes in the DNA damage marker γH2AX by FACS (Fig. 1D). The combination group exhibited a notable elevation of γH2AX levels compared to DMSO- and single agent-treated cells in both cell lines.

Collectively, our findings demonstrate the additive/synergistic effect of onvansertib and olaparib in ovarian cancer cell lines, leading to increased induction of G2/M arrest, DNA damage, and apoptosis.

### Antitumor activity of combined onvansertib and olaparib in ovarian cancer PDXs

Based on our in vitro data, six different PDX models were selected from our xenobank to study the in vivo antitumor activity of onvansertib alone and in combination with olaparib. All the PDXs were HGOCs, *TP53* mutated with both acquired or intrinsic olaparib resistance, as specified below and in Table 1 [34, 35]. MNHOC#22, MNHOC#266, and MNHOC#316DDP PDXs are i.p. transplanted models, while the others derived from s.c. transplanted ovarian carcinomas. Despite the different sites of injection, all the selected PDXs well reproduce the human pathology and are used for chemotherapeutic testing with reliable and reproducible results [35]. While MNHOC#124, MNHOC#239, and MNHOC#316DDP PDXs are *BRCA* wt and poorly responsive to PARPi treatment, MNHOC#218Ola is a subline generated in our laboratory after repeated in vivo cycles of olaparib treatment of the parental *BRCA1* mutated MNHOC#218 PDX originally very sensitive to olaparib. MNHOC#22 and MNHOC#266 PDXs (both *BRCA1* mutated) were derived from women treated with a platinum-based therapy [35], who were never treated with PARPi but yet resistant to olaparib and whose resistance mechanisms are under study.

**Table 1:** Characteristics of the selected PDX models.

PDX ID	DDP sensitivity	olaparib sensitivity	*TP53* status	*BRCA1* status	Site of tumor transplant
MNHOC#22	+++	-*	mut	mut	i.p.
MNHOC#266	+++	-*	mut	mut	i.p.
MNHOC#218Ola	+++	-^&^	mut	mut	s.c.
MNHOC#124	+++	-^$^	mut	wt	s.c.
MNHOC#239	++	-^$^	mut	wt	s.c.
MNHOC#316DDP	-	-^$^	mut	wt	i.p.

*+++* very sensitive, *++* sensitive, *−* resistant, * intrinsically resistant (whose mechanisms are under study), ^*&*^ acquired resistance,

^$^ resistant due to the presence of a wt *BRCA1,* DDP cisplatin, i.p. intraperitoneally, *s.c.* subcutaneously.

#### -BRCA1 mutated models

**Fig. 2:**
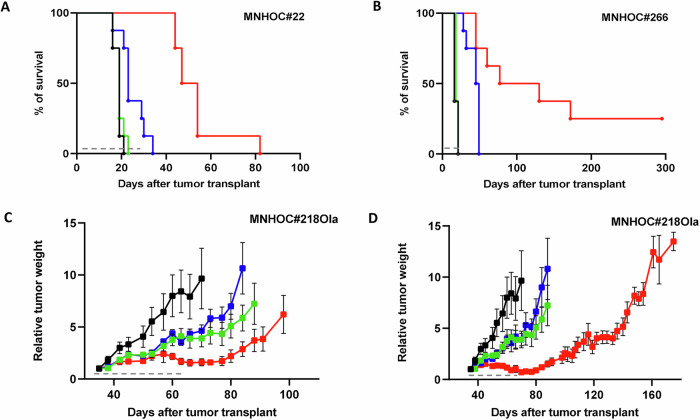
Antitumor activity of olaparib, onvansertib single agents and their combination in *BRCA1* mutated PDX models. **A** Survival curves and log-rank (Mantel–Cox) test in MNHOC#22 bearing mice. Tumor was transplanted intraperitoneally (i.p.) and at day 8 mice were randomized to receive vehicle (-●-), olaparib (100 mg/kg p.o. 5 days a week for 4 weeks, ), onvansertib (50 mg/kg p.o. 5 days a week for 4 weeks, ) and their combination (). *p* < 0.0023 onvansertib vs control; *p* < 0.0001: combo *vs* control and combo *vs* onvansertib. **B** Survival curves and log-rank (Mantel–Cox) test in MNHOC#266 bearing mice. MNHOC#266 PDX was transplanted i.p. and at day 8 mice were randomized to receive vehicle (-●-), olaparib (80 mg/kg p.o. 5 days a week for 4 weeks, ), onvansertib (50 mg/kg p.o. 5 days a week for 4 weeks, ) and their combination () *p* < 0.0001: combo *vs* olaparib; *p* < 0.0001: combo *vs* control; *p* < 0.0069 combo *vs* onvansertib. **C**, **D** MNHOC#218Ola xenografts were transplanted subcutaneously and grown until masses reached 100–150 mg. Mice were then randomized to receive vehicle (-●-), olaparib (80 mg/kg p.o. 5 days a week for 4 weeks, ), onvansertib (30 mg/kg in **C** or 45 mg/kg in **D** p.o. 5 days a week for 4 weeks, ), and their combination (). Data are the mean ± SEM of tumor masses, as described in Materials and Methods; each group consisted of 8 animals. Gray dashed lines represent treatment duration.

Mice transplanted intraperitoneally (i.p.) with MNHOC#22 and MNHOC#266 tumor cells were treated as specified in Material and Methods. Survival curves are reported in Fig. 2A, B. In both MNHOC#22 and MNHOC#266 olaparib as single agent was not active, as expected, while onvansertib displayed a statistically significant improvement of survival only in MNHOC#266 model. Conversely, the combination resulted in a threefold and ninefold increase in median survival compared to vehicle in the MNHOC#22 and MNHOC#266 models, respectively (Supplementary Table S2). In addition, two of the 8 mice transplanted with MNHOC#266 and treated with the combination were still tumor-free 250 days after tumor transplantation. As shown in Supplementary Fig. S4 the combination was well tolerated. Even though mice treated with the combination experienced greater body weight loss, than onvansertib single agent; however, all mice recovered after treatment withdrawal.

These data were corroborated in the MNHOC#218Ola s.c. model, where onvansertib was tested at two different doses (30 and 45 mg/kg) alone and in combination with 80 mg/kg olaparib (Fig. 2C, D and Supplementary Fig. S5). Again, single-agent treatments were largely ineffective, while the combinations of the two drugs displayed substantial tumor growth inhibition. The group treated with olaparib and the higher onvansertib dose (45 mg/kg) exhibited strong tumor regression during the 4-week treatment, followed by two weeks of tumor stabilization (Fig. 2C, D). Similar effects are also depicted in Supplementary Fig. S5, shown as log2 tumor reduction at the end of treatment.

#### -BRCA wt PDX models

**Fig. 3:**
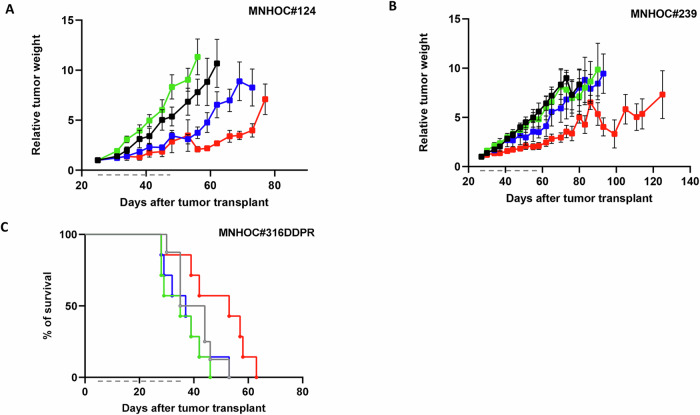
Antitumor activity of olaparib, onvansertib and their combination in *BRCA1* wt PDX models. **A** MNHOC#124 xenografts were transplanted subcutaneously, and mice were randomized when tumor masses reached 100-150 mg to receive vehicle (-●-), olaparib (100 mg/kg p.o. 5 days a week for 4 weeks, ), onvansertib (50 mg/kg p.o. 5 days a week for 4 weeks, ) and their combination (). Data are the mean ± SEM of tumor masses; each group consisted of 8 animals. **B** MNHOC#239 xenografts were transplanted subcutaneously and when tumor masses reached 100–150 mg, mice were randomized to receive vehicle (-●-), olaparib (100 mg/kg p.o. 5 days a week for 4 weeks, ), onvansertib (50 mg/kg p.o. 5 days a week for 4 weeks, ) and their combination (). Data are the mean ± SEM of tumor masses; each group consisted of 8 animals. **C** Log-Rank (Mantel-Cox) test in MNHOC#316DDPR. Tumor was transplanted and 8 mice were randomized to receive cisplatin (5 mg/kg i.v. once a week for 4 weeks, ), olaparib (100 mg/kg p.o. 5 days a week for 4 weeks, ), onvansertib (50 mg/kg p.o. 5 days a week for 4 weeks, ) and their combination (). *p* = 0.013, onvansertib vs control; *p* = 0.014, combo vs control; *p* = 0.045 combo vs onvansertib. Gray dashed lines represent treatment duration.

The therapeutic effect of the combination was further explored in PDXs models that were *BRCA1* wt, platinum-sensitive, and olaparib-resistant. Again, in the two models tested, the single agents were inactive or scarcely active, while the combination was quite effective (Fig. 3). Specifically, in MNHOC#124 model (Fig. 3A), olaparib treatment was completely inactive, a slight activity was observed with onvansertib treatment, while greater tumor growth inhibition could be observed in mice treated with the combination. Indeed, in this latter group all the mice (except one whose tumor never responded to the combination) showed disease stabilization during treatment, but tumors resumed growing soon after treatment withdrawal (Supplementary Figs. S6A and S7A). Similar observations were made with the MNHOC#239 model, for which the combination group showed the highest tumor growth inhibition again (Fig. 3B and Supplementary Figs. S6B and S7B).

Finally, we tested the combination in MNHOC#316DDP, a platinum/olaparib-resistant model generated in our laboratory by repeated in vivo cisplatin treatment of the parental MNHOC#316 platinum-sensitive PDX. The combination-treated mice had a statistically significant increase in survival time as compared to cisplatin or single agent-treated mice (Fig. 3C). Again, all the mice treated with the combination experienced body weight loss that was regained upon treatment withdrawal (Supplementary Fig. S8). Data obtained in this latter model suggest that the combination is not only active in olaparib-resistant PDXs, but is also potentially active in platinum-resistant PDXs.

Taken together, these data indicate that the combination of onvansertib and olaparib is well tolerated and exhibited significant efficacy in vivo in both platinum-sensitive and -resistant models. In addition, it also demonstrates the ability of onvansertib to counteract olaparib resistance, particularly in *BRCA* mutant models.

### Mechanistic basis for the activity of the onvansertib-olaparib combination

**Fig. 4:**
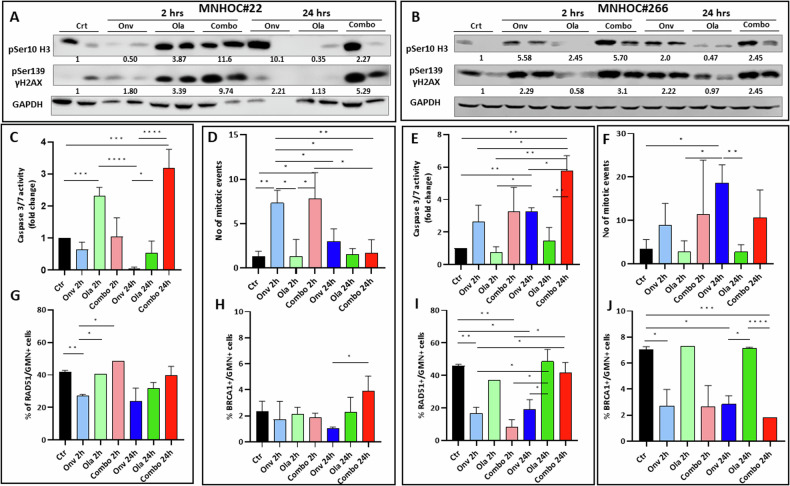
In vivo pharmacodynamic assessment of apoptosis, mitotic block, and DNA damage in PDXs treated or not with the single or combined drugs. **A**, **B** Western blot analysis showing pSer10 H3 and γH2AX protein levels in xenograft tumor protein extracts from MNHOC#22 (**A**) and MNHOC#266 (**B**). Two tumors from each group were used. Numbers below the western blot represents the fold changes of values in treated over control values. All the values were normalized over the GAPDH protein level. **C**, **E** Caspase 3/7 activity in tumor tissue extracts from treated mice of MNHOC#22 (**C**) and MNHOC#266 (**E**) (*n* = three biological replicates). **D**, **F** Number of mitotic events in MNHOC#22 (**D**) and MNHOC#266 (**F**) xenografts counted in three randomly selected fields at 400× of magnitude. **G**, **I** Percentage of RAD51/GMN+ cells in MNHOC#22 (**G**) and MNHOC#266 (**I**). **H**, **J** Percentage of BRCA1/GMN+ cells in MNHOC#22 (**H**) and MNHOC#266 (**J**). *p < 0.05; **p < 0.01; ***p < 0.001; ****p < 0.0001. Unpaired *t* test was used to test statistical differences.

To gain an understanding of the mechanism underlying the combinatorial effects of the drugs in vivo, we analyzed bona fide markers of DNA damage (γH2AX), cell death by apoptosis (γH2AX and caspase 3/7 activity) and cell cycle perturbation (pSer10 H3 levels and mitotic counts) in tumor extracts from MNHOC#22 and MNHOC#266 PDX bearing mice treated for 5 days with vehicle, single agents or the combination at the same doses used for antitumor activities. In MNHOC#22, pSer10 H3 increase could be observed in tumor treated with onvansertib at 24 h after the last treatment, at 2 h after olaparib treatment, but a higher increase could be observed in the combination treated tumors both a 2 and 24 h (Fig. 4A and Supplementary Fig. S9). At the same time, onvansertib caused almost no activation of γH2AX both at 2 and 24 h, olaparib induced an activation at 2 h that regressed by 24 h; the combined treatment caused a strong γH2AX at both timepoints. As regards MNHOC#266 model, slightly different results were observed, where an increase of both pSer10 H3 and γH2AX was observed at both timepoints in onvansertib treated tumors; olaparib treatment caused no increase of both markers at 24 h; combined treatment mimic the induction observed after onvansertib (Fig. 4B, Supplementary Fig. S9). Unfortunately, not always statistically significance differences were observed due to the biological variability among tumor samples. However, as a whole, these data would suggest in MNHOC#22 a greater induction of damage in the combined treatment versus single agents, while similar induction of damage in the combination and onvansertib-treated tumors in the MNHOC#266 model.

γH2XA is both a marker of DNA damage and induction of apoptosis [36], we explored the induction of apoptosis by evaluating caspase 3/7 activity in tumors from the different experimental groups. A trend toward higher caspase activities in the combination-treated mice was indeed observed at 24 h in both experimental models (Fig. 4C, E), statistically significant over controls and single olaparib treatment. In addition, a higher number of mitotic events, suggesting a block in M phase of the cell cycle, was observed in onvansertib and combination versus control vehicle-treated MNHOC#22 tumors only at 2 h (Fig. 4D), and an increasing number at both timepoints in MNHOC#266 tumors (Fig. 4F), not always reaching a statistical significance.

Considering the reported role of PLK1 in inhibiting DNA repair, including HR, we quantified the % of RAD51 foci-positive cells (RAD51/GMN+) in tumors of mice treated with single agents and with the onvansertib/olaparib combination. The two PDXs contain high levels of RAD51/GMN+ cells (>40%) at basal level, correlating with their resistance to olaparib [34]. Olaparib treatment did not cause any change in the % of RAD51/GMN+ cells, while onvansertib treatment caused a statistically significant decrease in both models at 2 h time point and a similar trend, not always statistically significant, at 24 h (Supplementary Fig. S10 and Fig. 4G, I). We also quantified the % of BRCA1 foci-positive tumor cells (BRCA1/GMN+ cells) in the same samples. The two PDXs exhibited a very low basal level of BRCA1/GMN+ cells, in line with the fact they harbor BRCA1 mutations. In MNHOC#22, we observed only a slight decrease of BRCA1/GMN + cells in onvansertib-treated mice at 24 h (Fig. 4H). This is likely due to the very low % of BRCA1/GMN+ cells in this PDX at the basal level (~2%) which renders difficult the measurement of a further significant reduction. In MNHOC#266 PDX, treatment with onvansertib, alone or combined with olaparib, reduced the % of BRCA1/GMN+ cells (Fig. 4J).

The decrease in RAD51 and BRCA1 positive cells in onvansertib-treated tumors could be related to the reported activity of PLK1 in HR and could partially explain the synergistic activity of the olaparib/onvansertib combination as a reduction in HR is expected to sensitize cells to PARPi, including olaparib. To further explore this hypothesis, we investigated the effect of onvansertib treatment on two DSB repair pathways using specific eGFP-reporter plasmids (HR-EGFP and NHEJ-GFP, respectively). Ovcar-5 cells were transfected along with a mix of I-*Sce*I meganuclease plasmid and HR and NHEJ specific plasmids, as detailed in Material and Methods. Interestingly, a 34 and 41% inhibition of HR and NHEJ-mediated DSB repair, respectively, could be observed in cells transfected and treated with onvansertib (40 nM, IC_50_ dose) for 24 h, compared to the cells transfected but not treated with onvansertib (Supplementary Fig. S11A, B), supporting a role of PLK1 in DNA repair. In addition, when U2OS cells stably transfected with the DR-GRF reporter [37] were transfected with SceI and treated with different dose of onvansertib, again an inhibition of HR repair was observed (Supplementary Fig. S11C).

The in vivo combination treatment significantly increased DNA damage when compared to single agents. The in vitro data indicated that onvansertib treatment effectively suppressed both HR and NHEJ repair mechanisms, which likely underlies the observed in vivo enhancement of DNA damage caused by the combination treatment.

### Gene expression changes induced by single and combined drug treatments in vivo

**Fig. 5:**
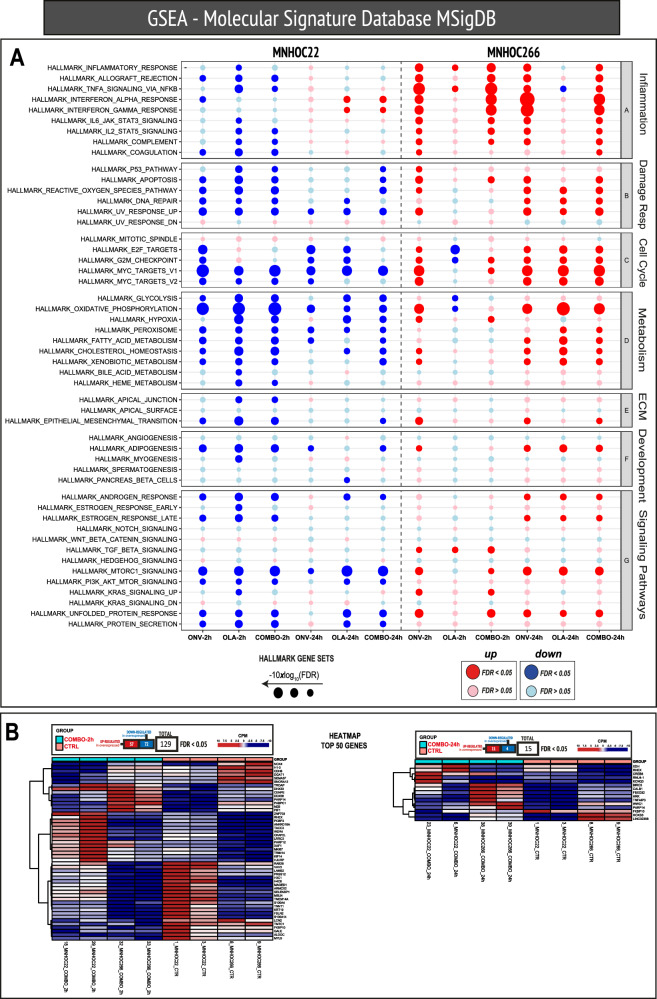
Differential transcriptomic analysis. **A** Dot plots of Hallmark gene sets indicating the regulation of the indicated pathways organized by the process. Each column represents the comparison listed at the bottom in the respective models. The size of each dot corresponds to the level of significance, whereby larger dots indicate greater significance of the gene set enrichment. The color of the dots represents the type of regulation: red for upregulation and blue for down-regulation. Only darkly colored dots are considered statistically significant, whereas dots with lighter colors are not statistically significant. **B** Heatmap of the significant genes of the ‘COMBO_2h vs CTR’ and ‘COMBO_24h vs CTR’ comparisons, respectively, considering the two models together.

To further explore the possible mechanism(s) underlying the striking activity observed with this combination, gene expression profiles were evaluated by RNAseq in MNHOC#22 and MNHOC#266 tumor samples derived from the single agents and the onvansertib/olaparib combination-treated mice. Figure 5 shows the gene expression analysis of the different experimental groups in the two PDX models, either 2 h or 24 h timepoints after 5 days of drug treatment. While in MNHOC#22 the main hallmark set gene pathways were downregulated after single agents and combination treatment, the same pathways were either not modified or upregulated in the MNHOC#266 model (Fig. 5A).

We looked for individual genes similarly regulated in the two PDX models at the two timepoints and found 129 (57 up- and 72 downregulated) and 15 (11 up- and 4 down-regulated) commonly regulated genes, at 2 h and 24 h, respectively (Fig. 5B). *FKBP10* (FKBP prolyl isomerase 10) was the only gene similarly downregulated at 24 h in the two models, while *RHEX* (regulator of haemoglobinization and erythroid cell expansion), *PARP14* (poly-ADP-ribose-polymerase family member 14) and *CREB5* (CAMP responsive element binding protein 5) were the genes commonly upregulated at 24 h (Fig. 5B).

No similar pathways were modulated following in vivo treatment with either single agents or the combination of two distinct *BRCA* mutated PDXs, and this divergence may be attributed to their different genetic background. Consequently, no specific pathway could be associated with the potent in vivo antitumor activity observed with the onvansertib/olaparib combination.

## Discussion

Olaparib has been approved as maintenance therapy for first-line and recurrent HGOC and for the treatment of platinum-sensitive relapsed HGOC, based on its tumor response rates and its ability to prolong the Progression-Free Survival in randomized clinical trials (for a recent review, see [38]). Resistance to olaparib has been described in vitro models [30] and is emerging in the clinical setting [39, 40]. Considering the widespread use of this drug, and of other PARPis, it is very likely that PARPi resistance will become an urgent clinical need in the near future. Therapeutic strategies to overcome PARPis resistance have been proposed and recently summarized [7, 41]. Here we report the antitumor activity of olaparib and onvansertib, a PLK1 inhibitor, in HR-proficient HGOC models with intrinsic or acquired resistance to olaparib.

One of the major determinants of olaparib sensitivity is the lack of functional HR, i.e. the inability to repair double-strand breaks in an error-free manner, and among the best-described mechanisms of olaparib resistance in an HR-deficient background is HR restoration [4]. PLK1 is a serine/threonine kinase with well-documented roles in entry into and progression through mitosis, and was also found to interfere with the DNA damage response pathway [12, 13, 17–19, 33]. Based on these considerations, we explored the combination of onvansertib and olaparib in different HGOC models with intrinsic and acquired resistance to olaparib. Olaparib single agent was almost inactive in all the models tested, while onvansertib single treatment displayed no or marginal antitumor activity in most of them. Conversely, the combination of onvansertib and olaparib was very active in all the ovarian cancer preclinical models tested, both in vitro and in vivo. The combined treatment at the doses used was well tolerated in vivo with a maximum body weight loss of 15%, which rapidly recovered after drug withdrawal. Neutropenia and thrombocytopenia have been reported to be the dose-limiting toxicity of onvansertib [21, 42], while olaparib myelotoxicity has been reported in 18–45% of treated patients [43, 44]; nevertheless at the doses used, we did not observe major toxicity issues, supporting that the two drugs can be safely administered.

The antitumor activity of the combination was particularly evident in olaparib-resistant PDX models harboring germline *BRCA1* pathogenic mutations (MNHOC#22, MNHOC#266, and MNHOC#218Ola). The mechanistic basis of olaparib resistance in these models is under investigation, however, likely involves the restoration of HR. A low level of RAD51-positive cells in proliferating tumors has been shown to be associated with HR deficiency [45] and to correlate with olaparib sensitivity in breast and ovarian carcinoma [34, 46]. Indeed, high basal levels of RAD51 foci-positive cells have been described in MNHOC#22 and MNHOC#266 and correlated with olaparib resistance [34]; similar data are available for MNHOC#218Ola. These data suggest that a restoration of HR occurred in these PDX models explaining the lack of olaparib sensitivity observed here and in previous studies [47]. However, the mechanisms involved in HR restoration are not clear, as no revertant mutations were found in *BRCA1* sequence in these PDX models.

Given the compelling responses in vivo, we sought to dissect the mechanisms underlying the potent activity of the combination. Indeed, in vitro experiments suggested that cells treated with the two drugs displayed a greater block in G2/M, higher induction of apoptosis, and increased levels of γH2AX, a biomarker of both apoptosis and DNA damage. Similar results were obtained in vivo in both MNHOC#22 and MNHOC#266 PDXs, even though a statistically significant difference was not always reached, probably due to the intrinsic tumor heterogeneity as compared to cell culture conditions. The G2/M block in cells treated with the onvansertib/olaparib combination could be the result of a critical interaction between PLK1 and PARP1 in regulating mitotic progression. In fact, PLK1 is a master regulator and coordinator of mitotic kinase signaling [9], and PARP1 has been reported to localize to the spindle and PARylate spindle proteins, i.e. microtubule-associated proteins, regulating spindle assembly and function [48]. The simultaneous inhibition of PLK1 and PARP1 could be responsible for the G2/M block favouring cell death through mitotic catastrophe. This hypothesis is corroborated by the reported antitumor activity of the PLK1 inhibitor onvansertib with different microtubule-targeting agents. Onvansertib was reported to exert antitumor activity in combination with the microtubule depolymerization inhibitor paclitaxel in mucinous ovarian cancer and HGOC [49] with abiraterone, due to its androgen receptor-independent effects on mitotic spindle in castrate-resistant prostate cancer models [29], and with the microtubule-targeting agents vincristine, vinblastine and eribulin in Ewing sarcoma [50]. As PLK1 is involved in the recovery from the G2 checkpoint after DNA damage and olaparib causes a G2 block [51], there is the possibility that when combined with olaparib, cells might experience a prolonged G2 arrest due to their inability to recover from the G2 checkpoint.

At the same time, inhibition of PLK1 activity during DNA repair response could potentiate DNA damage induced by PARPi and/or, create a temporary HR deficiency. Our data indicate that onvansertib is able to inhibit both HR and NHEJ repair pathways in vitro at nM concentrations, likely to be reached in vivo. Onvansertib also decreased the number of RAD51-positive tumor cells in vivo, indicative of reduced HR-mediated DNA repair. Synergistic cancer cell killing by the combination of olaparib and PLK1 inhibition, using other small molecules, including BI2536 and BI6727 (volasertib), has been reported in castration-resistant prostate cancer [52] and in HGOC cells with KRAS amplification [53]. In both experimental conditions, increased DNA damage/apoptosis by combination treatment was reported. While the former hypothesis would better fit in an HR-proficient background, both mechanisms could cooperate in an HR-deficient background, accounting for a better activity of the combination in this latter setting.

To further uncover the mechanisms underlying the antitumor effect of this combination, gene expression analyses by RNAseq were undertaken in two different PDX models at multiple timepoints following 5-day of treatment. Despite a striking in vivo antitumor activity of the combination in both PDX models, no common pathway modulation was found, suggesting that the disparate transcriptional profiles observed are likely to be dictated by the different genetic backgrounds of the two PDXs.

In conclusion, we report here the antitumor activity of the onvansertib/olaparib combination in vitro and in vivo using multiple olaparib-resistant models of ovarian carcinoma that include both *BRCA* mutated and WT genetic backgrounds. While onvansertib single agent was partially active in the models tested, in all cases, PLK1 inhibition was able to overcome olaparib resistance. Considering that the combination was well tolerated, these experimental data support and foster the clinical evaluation of onvansertib with PARPi, particularly in HGSOC PARPi-resistant settings.

## Methods and materials

### Cell lines and drug treatment

Cell lines were obtained from ATCC or DSMZ, and their authentication has been carried out by the authors within the last 6 months. Cells were cultured in RPMI (Ovcar-3, Ovcar-5), McCoy’s5a (ES-2), or DMEM (ID8) supplemented with 10%FBS and 2 mM L-glutamine. All cell lines were regularly tested for mycoplasma contamination. Cells were treated with olaparib (Targetmol) and onvansertib (provided by Cardiff Oncology) at the indicated concentrations.

### Cell viability, cell cycle analyses, and apoptosis

Growth curves were obtained by seeding the cells in 96-well or 384-well plates, and proliferation was examined with either the MTS assay (Promega, Madison, WI, USA) or the CellTiterGlo® assay (Promega) per the manufacturer’s instructions. Drug synergy was analyzed with Combenefit software using the BLISS algorithm [54]. Ovcar-3 and ES-2 cells were stained for flow cytometry analyses with the mitotic marker phospho-histone H3 (Ser28), the apoptotic marker cleaved-caspase 3, or the DNA damage/apoptotic marker yH2AX as specified in Supplementary Materials.

### In vivo antitumor activity

The factorial experimental design of our in vivo studies was processed by a biostatistician. All the in vivo work was conducted as specified in Supplementary Materials upon approval by then institutional review board, and the Italian Ministry of Health approved all the in vivo experiments performed with PDXs (approval number no 475/2017-PR).

### Western blot analysis

Western blot analyses were done according to standard procedures, specified in Supplementary Materials.

### Caspase 3/7 activity assay

Caspase 3/7 activity was measured by the Caspase-Glo®3/7 kit (Promega) following manufacturer instructions. Briefly, protein extracts were transferred in a white 96-well plate in duplicate for each sample, and the Caspase-Glo reagent was then added to all samples and incubated at 37 °C and after 45 min luminescence was read by GloMax® Microplate Reader (Promega). Caspase activity was expressed as mean relative light units normalized to the protein concentration.

### Mitotic events

Histological evaluation (hematoxylin and eosin staining) was carried out to detect mitosis in cytopellets from peritoneal effusion of mice inoculated intraperitoneally with tumoral cells. Mitotic events were counted in blinded conditions on three randomly selected histological fields at 400×.

### Immunofluorescence (IF) detection of nuclear foci on formalin-fixed paraffin-embedded samples

To quantify RAD51 and BRCA1 nuclear foci, we used an IF-based method, as previously described [45, 46] and specified in Supplementary Materials. RAD51 or BRCA1 foci were quantified by blinded scoring of the percentage of geminin (GMN)-positive tumor cells with five or more foci per nucleus (named RAD51/GMN+ or BRCA1/GMN+, respectively). At least 100 GMN-positive cells in ten different areas of the section were analyzed (Supplementary Fig. S11).

### DNA repair assay

Pathway-specific DSB repair efficiencies were investigated by using a functional green fluorescent protein (eGFP)-based assay [55] and specified in Supplementary Materials.

### RNAseq

Peritoneal tumor cells recovered by peritoneal lavage were immediately snap-frozen, and then further processed as detailed in Supplementary Materials.

### Statistical analysis

In vitro experiments were replicated at least three times unless otherwise indicated, and the data were expressed as mean ± standard deviation or as mean ± standard error of the mean.

Differences between groups were analyzed with unpaired Student’s *t* test, one-sided or two-sided paired tests. Differences were considered statistically significant for *p* < 0.05. Sample sizes were chosen based on preliminary results to ensure a power of 80% and an alpha level of 5%. No data or animals were excluded from the analyses. Statistical significance was determined with GraphPad Prism 9 (GraphPad Software).

### Supplementary information

Supplementary Materials

Supplementary Figures

Supplementary Figure 12

## Data Availability

All the in vitro and in vivo data generated and analyzed during this study are available from the corresponding author upon reasonable request. The raw RNAseq data are available in the Annotare database EMBL-EBI (https://www.ebi.ac.uk/fg/annotare/) under the accession numbers: E-MTAB-1305.
