# Supplementary material for: Onvansertib treatment overcomes olaparib resistance in high-grade ovarian carcinomas
Source: Cell Death Dis. 2024 Jul 22;15(7):521. doi: 10.1038/s41419-024-06894-1 (PMC11263393; doi:10.1038/s41419-024-06894-1)
Supplement: Supplementary file 1 — Supplementary Materials [file 41419_2024_6894_MOESM1_ESM.docx]

**Supplementary Materials**

Onvansertib treatment overcomes olaparib resistance in high grade ovarian carcinomas

Michela Chiappa^1^, Alessandra Decio^2^, Luca Guarrera^3^, Ilaria Mengoli^1^, Anju Karki^4^, Divora Yemane ^4^, Carmen Ghilardi^2^, Eugenio Scanziani^5,6^, Simone Canesi^5,6^, Maria C. Barbera^3^, Ilaria Craparotta^3^, Marco Bolis^3^, Robert Fruscio^7^, Chiara Grasselli^8^, Tommaso Ceruti^9^, Massimo Zucchetti^9^, Jesse C Patterson^10^, Lu Robin A^10^, Yaffe Micheal B^10^, Maya Ridinger^4^, Giovanna Damia^1^* and Federica Guffanti^1^

**Materials and Methods**

**Flow cytometry analyses.** For cell cycle analyses, cells were fixed with BD Cytofix^TM^ (BD Biosciences) for 15 minutes and stained with DAPI. For intracellular stains, cells were fixed (BD Cytofix^TM^, 15 minutes), permeabilized (BD Phosflow™ Perm Buffer III, 30 minutes), and stained with antibodies (Supplementary Table S1) and 1 µg/mL DAPI (BD Biosciences). FACSCelesta^TM^ (BD Biosciences) was used for flow cytometry analysis and the data were processed using FlowJo.

***In vivo antitumor activity.*** Five-week-old female NCr-nu/nu mice were obtained from Envigo Laboratories (Italy) and maintained under specific pathogen-free conditions, housed in isolated vented cages, and handled using aseptic procedures. Tumor fragments were subcutaneously (s.c.) transplanted or tumor cell suspensions (approximately 10-15x10^6^ cells/mouse) were orthotopically (intraperitoneally, i.p.) implanted. When s.c. engrafted PDXs reached approximately 100-150 mg of tumor weight (TW), mice were randomized (8 mice/group) to receive vehicle or specific treatments (olaparib, onvansertib or combination of the two), while i.p. xenografts were treated 7-10 days after transplant. Onvansertib dissolved in 0.5% methylcellulose solution with 0.1% of Tween-80 and given orally (p.o.) at the dose of 30-50 mg/kg daily, 5 days a week for 4 weeks. Olaparib (Targetmol) was dissolved in 10% v/v dimethylsulfoxide (DMSO), 10% w/v HP-beta-cyclodestrine and diluted in sterile water, and given orally at the dose of 80-100 mg/kg daily, 5 days a week for 4 weeks, two hours after onvansertib treatment. Tumor growth was measured twice a week with a Vernier caliper, and tumor weights (1mg = 1mm^3^) were calculated as follows: (length [mm] × width^2^ [mm^2^])/2, where width<length); body weights were recorded and considered an indirect parameter of drug toxicity. For i.p. transplanted tumors, mice were weighed three times a week and the appearance of ascites was recorded. A blinding method was adopted during each measurement of tumor volume. The person who routinely measured and register tumor volume was not aware of the treatment received by the animals. For s.c. PDXs, treatment efficacy was expressed as best tumor growth inhibition (T/C), calculated as follows: T/C%= [(mean TW treated mice/ mean TW control mice) x100]. Treatment efficacy in orthotopically transplanted PDXs was expressed as increase in lifespan (ILS), calculated as: ILS%= [(median survival days of treated mice– median survival days of control mice)/ median survival days of treated mice]x100. For pharmacodynamic studies, three tumor bearing mice for each experimental group were treated for 5 days with the single or combined drugs following the schedules used in antitumor activity studies and then euthanized at 2 and 24h after the last dose of onvansertib. Tumors were removed and in part snap-frozen and in part fixed in formalin and then paraffin embedded (FFPE) for further analyses.

***Western blot analysis.*** Cell pellets were lysed for 30 min in ice-cold whole cell extract buffer (50 mM TrisHCl pH 7.4, 250 mM NaCl, 0.1% Nonidet NP40, 5 mM EDTA, 50 mM NaF and a protease inhibitor cocktail (Sigma- Aldrich)). Lysates were cleared by centrifuging at 12,000 rpm for 15 min and the protein concentration was determined using a Bio-RAD assay kit (BIO-RAD Laboratories S.r.l). Tumor lysates (40 µg) were resolved on 10-12% SDS-PAGE (polyacrylamide gel electrophoresis) gels. Proteins were then transferred to nitrocellulose membranes (Merck Millipore). Immunoblotting was done with the antibodies specified in Supplementary Table S1. The secondary antibodies conjugated with horseradish peroxidase (HRP) anti-rabbit #1706515 and anti-mouse #1706516 were purchased from BIO-RAD Laboratories. Horseradish peroxidase (HRP) substrate (ECL Western Blotting Detection, Amersham-Life Science) was added and the signal was detected with the Odyssey Fc instrument (Li-COR). Cell lysates were analyzed by Western-Blot using the Wes System (ProteinSimple), a combination of capillary electrophoresis and immunodetection techniques, following the manufacturer’s protocols. Primary and secondary antibodies are listed in Supplementary Table S1. Quantitative analysis was performed using Compass Software (ProteinSimple). Uncropped version of gels are shown in Figure S12.

***Immunofluorescence (IF) detection of nuclear foci on FFPE samples.*** To quantify RAD51 nuclear foci, we used an IF-based method (1, 2). FFPE PDX tissue sections were deparaffinized and antigens were retrieved with DAKO Antigen Retrieval Buffer pH 9.0 (Agilent DAKO). The primary and secondary antibodies used to detect RAD51, BRCA1 and geminin in IF are listed in Supplementary Table S1. Nuclei were stained with 4’,6-diamidino-2-phenylindole (DAPI) (30 ng/mL in PBS, Sigma-Aldrich). Slides were mounted with Vectashield solution (VectorLab). Slices were observed using the ECLIPSE Ti2-E (Nikon) fluorescence microscope, with the 60x/1.27 WI Plan APO IR, ∞ 0.15/0.19 WD 0.18-0.16 objective (Nikon). RAD51 and BRCA1 foci were quantified by scoring in blind the percentage of geminin-positive tumor cells with 5 or more foci per nucleus (named RAD51/GMN+ and BRCA1/GMN+ cells, respectively). At least 100 geminin-positive tumor cells in three different areas of the tissue section were analyzed.

***DNA repair assays***. Two distinct DNA repair assays have been performed to investigate pathway-specific double strand breaks (DSBs) repair efficiencies. The first is a functional eGFP-based assay that have been previously described (3). In detail, Ovcar-5 cells were co-transfected with 10µg of different plasmid mixtures containing the meganuclease expression plasmid (pCMV-I-Sce1) together with one of the recombination substrates (HR-eGFP/3′eGFP and tot-NHEJ) and the wt eGFP plasmid for the determination of transfection efficiency (4, 5). One million cells were seeded in 6-well plates and after 24h were transfected using Lipofectamine 2000 (Invitrogen), following manufacturer instructions. Cells treated with Lipofectamine 2000 solution but not transfected with plasmids, were used as negative control. Samples were performed in duplicate to compare untreated cells and cells treated with onvansertib at the dose of IC_50_ (40nM) under identical transfection conditions. Onvansertib was added in the culture medium immediately after transfection. After 24h, cellular fluorescence was quantified by flow cytometry (Cytoflex LX, Beckman Coulter) and analysis was performed using Kaluza software version 2.1 (Beckman Coulter). Recombination frequencies were normalized for transfection efficiency based on the fraction of wt eGFP positive cells.

The second test relies on the use of U2OS-DR-GFP cells, which contain a chromosomally integrated DR-GFP assay to measure HR repair efficiency (6)[. Differently from the above-mentioned assay, this system does not require the co-transfection with multiple plasmids, but only with 2 µg of](https://doi.org/10.1007/978-1-61779-129-1_16) pCMV-I-Sce1 plasmid, while seeding and transfection conditions were maintained similar as previously described. Samples were performed in triplicate, treated or not with onvansertib at the doses of 40nM and 80nM after lipofectamine transfection, while cells treated only with lipofectamine solution without plasmid were used as negative controls. HR repair activity was assessed 24h after treatment by quantification of the percentages of GFP positive cells compared with untreated cells, using flow cytometry. These assays were repeated at least twice.

***RNAseq.*** Before library preparation, RNA concentration was evaluated through Qubit™ RNA High Sensitivity Assay Kit (Invitrogen) while RNA quality was established using 4200 Tapestation (Agilent Technologies). Following the TruSeq Stranded Total RNA protocol (Illumina), at least 500 ng of RNA whose RIN value was between 7 and 9, were used for RNA sequencing. RNA Sequencing (RNA-Seq) was run on a NextSeq 500 sequencer (Illumina) using a 1x75 high-output flow cell with 13 samples/run. The overall quality of sequencing reads was evaluated using FastQC (v.0.11.9) (7). Sequence alignments of total-RNA (stranded) to the combined reference of the mouse and human genomes (GRCm39 and GRCh38) were performed using STAR (v.2.7.9a) in two-pass mode (8). Subsequently, the fraction corresponding to the human genome was filtered and selected for the execution of further analysis. Gene expression was quantified at the gene level by using the comprehensive annotations made available by Gencode (V38 GTF File). Samples were adjusted for library size and normalized with the variance stabilizing transformation (vst) in the R statistical environment using DESeq2 (v1.28.1) pipeline (9). GSEAs were performed using the limma (v.3.44.3) package. Gene-set collections were retrieved from the Molecular Signature Database (MSigDB) (10). P-values were corrected for multiple testing using the false discovery rate (FDR) procedure, with the significance threshold set to 0.05. The raw data are available in the Annotare database EMBL-EBI (<https://www.ebi.ac.uk/fg/annotare/>) under the accession numbers: E-MTAB-13055.

**Supplementary Table S1. List of the antibodies used in this study.**

| **Target** | **Host species** | **Dilution** | **Product Name** | **Company** |
| --- | --- | --- | --- | --- |
| **RAD51** | Rabbit monoclonal | 1:1000 (IF) | ab133534 | Abcam |
| **Geminin** | Mouse monoclonal | 1:100 (IF) | GEMININ-L-CE | NovoCastra |
| **BRCA1** | Mouse monoclonal | 1:50 (IF) | sc-6954 | Santa Cruz Biotechnology |
| **Geminin** | Rabbit polyclonal | 1:400 (IF) | Geminin Polyclonal antibody  10802-1-AP | ProteinTech Group |
| **anti-mouse** | Goat polyclonal | 1:500 (IF) | Goat anti-Mouse IgG (H+L) Cross-Adsorbed Secondary Antibody, Alexa Fluor™ 488 | Thermo Fisher  Scientific |
| **anti-mouse** | Goat polyclonal | 1:500 (IF) | Goat anti-Mouse IgG (H+L) Cross-Adsorbed Secondary Antibody, Alexa Fluor™ 568 | Thermo Fisher  Scientific |
| **anti-rabbit** | Goat polyclonal | 1:500 (IF) | Goat anti-Rabbit IgG (H+L) Cross-Adsorbed Secondary Antibody,  Alexa Fluor™ 488 | Thermo Fisher  Scientific |
| **anti-rabbit** | Goat polyclonal | 1:500 (IF) | Goat anti-Rabbit IgG (H+L) Cross-Adsorbed Secondary Antibody,  Alexa Fluor™ 568 | Thermo Fisher  Scientific |
| **pSer28 H3** | Rat monoclonal | 1:200 (FACS) | #641003 | Biolegend |
| **pSer10 H3** | Rabbit polyclonal | 1:500 (WB) | #06-570 millipore | Millipore Sigma |
| **pSer139 γH2AX** | Rabbit monoclonal | 1:500 (WB)  1:50 (PS) | #9718 | Cell Signaling |
| **pSer139 γH2AX** | Rabbit monoclonal | 1:50 (FACS) | FITC anti-Phospho-Histone H2A.X (Ser139) - #613404 | Biolegend |
| **GAPDH** | Goat polyclonal | 1:500 (WB) | sc-20357 | Santa Cruz Biotechnology |
| **Cyclin B1** | Rabbit monoclonal | 1:150 (PS) | #12231 | Cell Signaling |
| **Vinculin** | Mouse monoclonal | 1:500 (PS) | sc-25336 | Santa Cruz Biotechnology |
| **Cleaved-casp3** | Rabbit monoclonal | 1:50 (PS, FACS) | #9664 | Cell Signaling |
| **Cleaved-PARP** | Rabbit monoclonal | 1:50 (PS) | #5625 | Cell Signaling |
| **β-Actin** | Mouse monoclonal | 1:100 (PS) | sc-47778 HRP | Santa Cruz Biotechnology |
| **anti-rabbit**  **(for β-Actin)** | Goat polyclonal | 1:2 (PS) | HRP Conjugate: #042-206 | Bio-Techne |
| **anti-mouse** | Goat polyclonal | 1:2 (PS) | HRP Conjugate: # 042-205 | Bio-Techne |
| **anti-rabbit** | Goat polyclonal | 1:3000 (WB) | HRP Conjugate #1706515 | BIO-RAD  Laboratories S.r.l. |
| **anti-mouse**  **anti-rabbit** | Goat polyclonal | 1:3000 (WB) | HRP Conjugate #1706515 | BIO-RAD Laboratories. |
| **anti-goat**  **anti-mouse** | Mouse polyclonal | 1:2000 (WB) | HRP Conjugate #1706516 | Santa Cruz Biotechnology |
| **anti-goat** | Mouse policlonal | 1:2000 (WB) | sc-2354 | Santa Cruz Biotechnology |

IF: immunofluorescence, WB: Western-Blot, PS: ProteinSimple

**Supplementary Table S2. Median survival and best tumor growth inhibition or increase in life span in the different PDXs.**

| **PDX ID** | **Controls median (days)** | **Onvansertib median (days)** | **Olaparib median (days)** | **Combination median**  **(days)** | **T/C or ILS Onvansertib** | **T/C or ILS Olaparib** | **T/C or ILS Combination** |
| --- | --- | --- | --- | --- | --- | --- | --- |
| **MNHOC#22** | **19** | **23** | **19** | **52** | **21** |  | **173** |
| **MNHOC#266** | **16** | **47** | **18** | **103** | **194** | **12,5** | **543,8** |
| **MNHOC#218Ola** | **80** | **99** | **112** | **179** | **39** | **45** | **7** |
| **MNHOC#124** | **73** | **80** | **56** | **98** | **44** | **84** | **22** |
| **MNHOC#239** | **84,5** | **91,5** | **96** | **129** | **62** |  | **41** |
| **MNHOC#316DDP** | **39,5** | **38** | **37** | **57** | **0** | **0** | **44** |

T/C tumor growth inhibition (treated tumor weight mean/control tumor weight mean); ILS: increase in life span ((treated median survival time-control median survival time)/ control median survival time).

**Legends to Supplementary Figures**

**Supplementary Figure S1. Onvansertib and olaparib combination in ID8 murine cell lines proficient and deficient in *BRCA1***. **Panel** **A.** Dose-response curve of olaparib in ID8-F3 (upper panel) and its subline made resistant to olaparib (Ola) (lower panel) as single agent and in combination with different onvansertib (ONV) doses. Data represent the mean ± standard deviation (SD) of three independent experiments done in sextuplicate. **Panel B.** Dose-response curve of olaparib in ID8 BRCA1-/- (upper panel) and its subline made resistant to olaparib (lower panel) as single agent and in combination with different onvansertib doses. Data represent the mean ± SD of three independent experiments done in sextuplicate. Tables on the right represent the olaparib IC_50_s alone or in combination with the corresponding onvansertib dose.

**Supplementary Figure S2. Combination of onvansertib and other PARPi in human Ovcar-3 cells.** Ovcar-3 cells were treated for 6 days with increasing doses of onvansertib and the PARP inhibitors niraparib (left) or AZD5305 (right). Drug synergy is indicated by blue squares within the Bliss Synergy Heatmap.

**Supplementary Figure S3. Onvansertib and olaparib combination in human ovarian cancer cells.** Ovcar-3 and ES-2 cells were respectively treated with DMSO, onvansertib (30nM and 50nM) olaparib (2.5µM and 5μM), or the combination (Onv+Ola) and at the indicated time points fixed, cyclin-B1 (panel A), cleaved-caspase-3 (cl-casp3) and cleaved-PARP (cl-PARP) (panel B) protein levels were evaluated by Western Blot analysis. β-Actin and Vinculin were used as protein loading controls.

**Supplementary Figure S4. Relative body weight in MNHOC#22 and MNHOC#266 bearing mice.** Mice transplanted with MNHOC#22 (left panel) and MNHOC#266 (right panel) were treated as described in material and methods with vehicle (CTRL, black), olaparib 80-100 mg/kg (OLA, green), onvansertib 50 mg/kg (ONV, blue) and their combination (COMBO, red). Mice body weights were recorded every three days. Data are expressed as the relative body weight and represent the mean ± SEM of 8-10 mice per group.

**Supplementary Figure S5. Antitumor activity of onvansertib, olaparib and their combination in MNHOC#218Ola model.** Log2 tumor weight (tumor at the end of treatment (T28) - tumor at the beginning of treatment (T0)) in MNHOC#218Ola-bearing mice treated with vehicle (CTRL), olaparib 80mg/kg (OLA), onvansertib 30 or 45 mg/kg (ONV) and their combination (COMBO). Unpaired *t-test* was applied for statistical analyses.

**Supplementary Figure S6.** **Antitumor activity of onvansertib, olaparib and their combination in MNHOC#124 and MNHOC#239 models**. Tumor growth curves of individual mice bearing MNHOC#124 PDX (panel A) and MNHOC#239 PDX (panel B). MNHOC#124 xenograft was transplanted subcutaneously and when tumor masses reached 100-150 mg, mice were randomized to receive vehicle (CTRL), olaparib (OLA, 100 mg/kg), onvansertib (ONV, 50 mg/kg) and their combination (COMBO). MNHOC#239 xenograft was transplanted subcutaneously and when tumor masses reached 100-150mg, mice were randomized to receive vehicle (CTRL), olaparib (OLA, 80 mg/kg), onvansertib (ONV, 40 mg/kg) and their combination (COMBO). Graphs represent the tumor weight (mg) of each single mouse, in the four different groups.

**Supplementary Figure S7.** **Antitumor activity of onvansertib, olaparib and their combination in MNHOC#124 and MNHOC#239 models**. **Panel A.** **MNHOC#124.** Log2 tumor weight (tumor at the end of treatment (T28) - tumor at the beginning of treatment (T0)) in MNHOC#124-bearing mice treated with vehicle (CTRL), olaparib (OLA, 80 mg/kg), onvansertib (ONV, 40 mg/kg) and their combination (COMBO). **Panel B.** **MNHOC#239.** Log2 tumor weight (tumor at the end of treatment (T28) - tumor at the beginning of treatment (T0)) in MNHOC#239-bearing mice treated with vehicle (CTRL group), olaparib (OLA, 100 mg/kg), onvansertib (ONV, 50 mg/kg) and their combination (COMBO group). Each group consisted of 8 animals..

**Supplementary Figure S8. Relative body weight of MNHOC#316DDP bearing mice.** Mice transplanted with MNHOC#316DDP were treated as described in material and methods with cisplatin (DDP) as control group, olaparib (OLA, 80 mg/kg), onvansertib (ONV, 40 mg/kg) and their combination (COMBO). Mice body weights were recorded every three days. Data are expressed as the relative body weight and represent mean ± SEM of 8 mice per group.

**Supplementary Figure S9. Densitometric quantification of pSer10-H3 and pSer139-H2AX western blot results.** Densitometric analysis of by western blot of pSer10-H3 and **pSer139-**H2AX protein levels in MNHOC#22 (upper panel) and MNHOC#266 PDXs (lower panel), treated with vehicle (CTRL), onvansertib (ONV), olaparib (OLA) and their combination (COMBO) at 2 and 24h time points. Data are the mean ± SD normalized over GAPDH protein levels. Unpaired *t-test* was applied for statistical analyses and only statistically significant differences are shown in the Figure.

**Supplementary Figure S10. Percentage of homologous recombination (HR) and non-homologous end joining (NHEJ) repair in Ovcar-5 and U2OS-DR-GFP cells treated with onvansertib**. **Panel A**: Ovcar-5 cells were transfected along with a mixture of I-*Sce*I meganuclease plasmids and HR and NHEJ specific plasmids, respectively, and treated or not for 24h with onvansertib after transfection (detailed in material and methods). Data are expressed as inhibition of HR and NHEJ mediated DSBs repair in cells transfected and treated with onvansertib (40 nM, IC_50_ dose) for 24h, compared to the cells transfected but not treated with onvansertib. Data represent mean ± SEM of three experiments done in triplicate. **Panel B:** % of DSB repair frequency in Ovcar-5 cells treated or not with two different doses of onvansertib (ONV 40nM, IC_50_). Data are the mean ± SEM of two experiments done in triplicate. Two-way ANOVA was used for statistical analysis. **Panel C:** U2OS-DR-GFP cells were transfected with I-*Sce*I meganuclease plasmids and treated or not with two doses of onvansertib (40nM and 80nM) for 24h before FACS analysis (see material and methods section). The reduced % of cells positive for the GFP over the control group (transfected but not treated with onvansertib), readout of the inhibition of homologous recombination capacity to repair DSBs induced by I-*Sce*I, was calculated and plotted. One-way ANOVA was used for statistical analysis.

**Supplementary Figure S11. RAD51 and BRCA1 foci positive and negative cells.** Representative images of immunofluorescent RAD51 foci and BRCA1 foci positive and negative cells in MNHOC#22 and MNHOC#266 PDXs. Cancer cell nuclei are stained with DAPI in blue, geminin in green and nuclear foci are red dots. White arrows point RAD51/GMN+ and BRCA1/GMN+ nuclei, expressing both geminin and at least 5 foci per nucleus. Magnitude 60x.

**Supplementary Figure S12. Uncropped western blots shown in Figure 4.**

**References**

1. Castroviejo-Bermejo M, Cruz C, Llop-Guevara A, Gutierrez-Enriquez S, Ducy M, Ibrahim YH, et al. A RAD51 assay feasible in routine tumor samples calls PARP inhibitor response beyond BRCA mutation. EMBO Mol Med. 2018;10(12).

2. Cruz C, Castroviejo-Bermejo M, Gutierrez-Enriquez S, Llop-Guevara A, Ibrahim YH, Gris-Oliver A, et al. RAD51 foci as a functional biomarker of homologous recombination repair and PARP inhibitor resistance in germline BRCA-mutated breast cancer. Ann Oncol. 2018;29(5):1203-10.

3. Deniz M, Kaufmann J, Stahl A, Gundelach T, Janni W, Hoffmann I, et al. In vitro model for DNA double-strand break repair analysis in breast cancer reveals cell type-specific associations with age and prognosis. FASEB J. 2016;30(11):3786-99.

4. Akyuz N, Boehden GS, Susse S, Rimek A, Preuss U, Scheidtmann KH, et al. DNA substrate dependence of p53-mediated regulation of double-strand break repair. Mol Cell Biol. 2002;22(17):6306-17.

5. Bennardo N, Gunn A, Cheng A, Hasty P, Stark JM. Limiting the persistence of a chromosome break diminishes its mutagenic potential. PLoS Genet. 2009;5(10):e1000683.

6. Nakanishi K, Cavallo F, Brunet E, Jasin M. Homologous recombination assay for interstrand cross-link repair. Methods Mol Biol. 2011;745:283-91.

7. Andrews SBB-FAQCtfHTSDhwbbaupf. 2010.

8. Dobin A, Davis CA, Schlesinger F, Drenkow J, Zaleski C, Jha S, et al. STAR: ultrafast universal RNA-seq aligner. Bioinformatics. 2013;29(1):15-21.

9. Love MI, Huber W, Anders S. Moderated estimation of fold change and dispersion for RNA-seq data with DESeq2. Genome Biol. 2014;15(12):550.

10. Liberzon A, Birger C, Thorvaldsdottir H, Ghandi M, Mesirov JP, Tamayo P. The Molecular Signatures Database (MSigDB) hallmark gene set collection. Cell Syst. 2015;1(6):417-25.
