# Supplementary material for: Onvansertib treatment overcomes olaparib resistance in high-grade ovarian carcinomas
Source: Cell Death Dis. 2024 Jul 22;15(7):521. doi: 10.1038/s41419-024-06894-1 (PMC11263393; doi:10.1038/s41419-024-06894-1)
Supplement: Supplementary file 2 — Supplementary Figures [file 41419_2024_6894_MOESM2_ESM.pptx]

## Slide 1
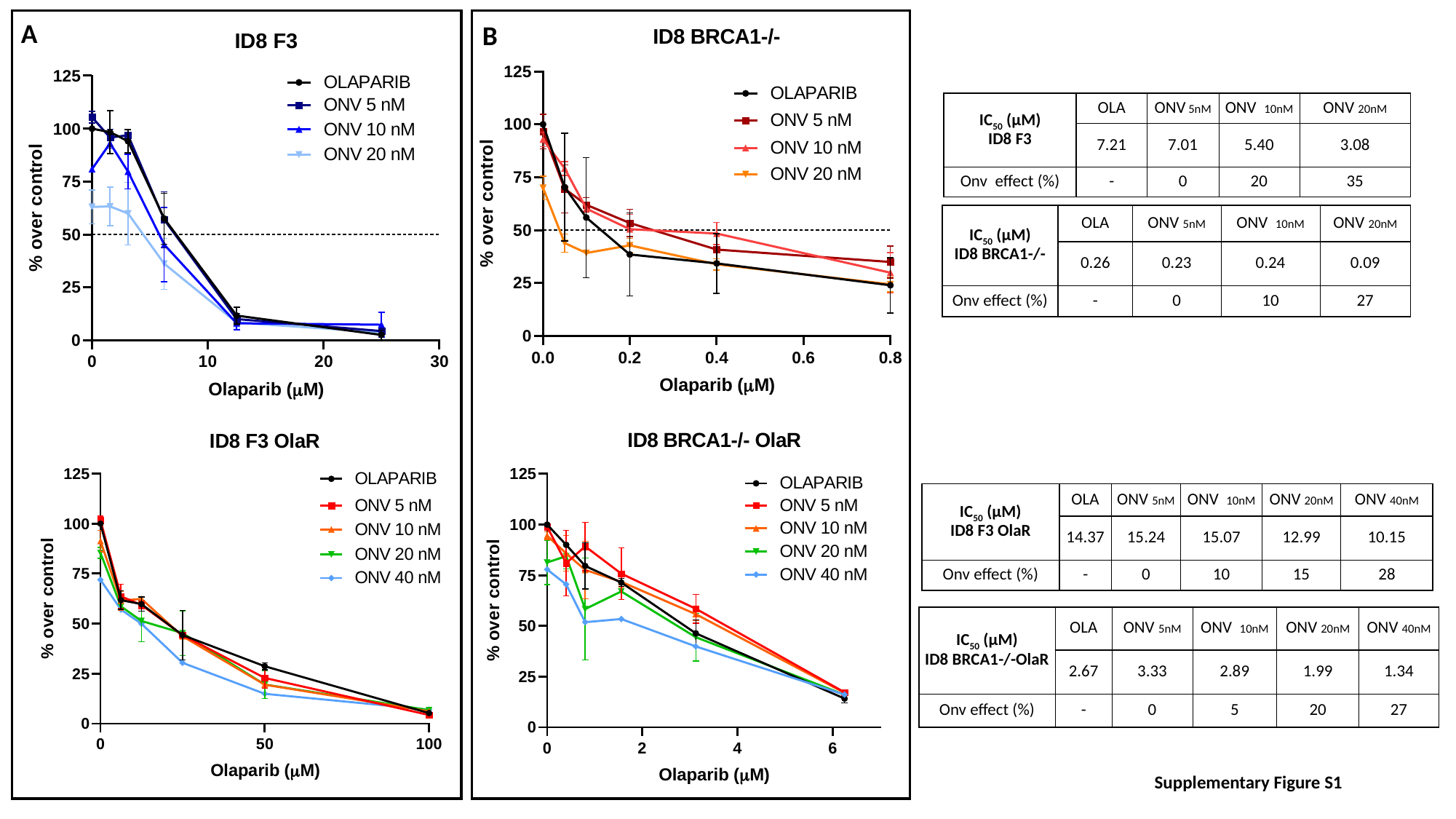

A
B
| IC50 (µM) ID8 F3 | OLA | ONV 5nM | ONV 10nM | ONV 20nM |
| --- | --- | --- | --- | --- |
| | 7.21 | 7.01 | 5.40 | 3.08 |
| Onv effect (%) | - | 0 | 20 | 35 |
| IC50 (µM) ID8 BRCA1-/- | OLA | ONV 5nM | ONV 10nM | ONV 20nM |
| --- | --- | --- | --- | --- |
| | 0.26 | 0.23 | 0.24 | 0.09 |
| Onv effect (%) | - | 0 | 10 | 27 |
| IC50 (µM) ID8 F3 OlaR | OLA | ONV 5nM | ONV 10nM | ONV 20nM | ONV 40nM |
| --- | --- | --- | --- | --- | --- |
| | 14.37 | 15.24 | 15.07 | 12.99 | 10.15 |
| Onv effect (%) | - | 0 | 10 | 15 | 28 |
| IC50 (µM) ID8 BRCA1-/-OlaR | OLA | ONV 5nM | ONV 10nM | ONV 20nM | ONV 40nM |
| --- | --- | --- | --- | --- | --- |
| | 2.67 | 3.33 | 2.89 | 1.99 | 1.34 |
| Onv effect (%) | - | 0 | 5 | 20 | 27 |
Supplementary Figure S1

## Slide 2
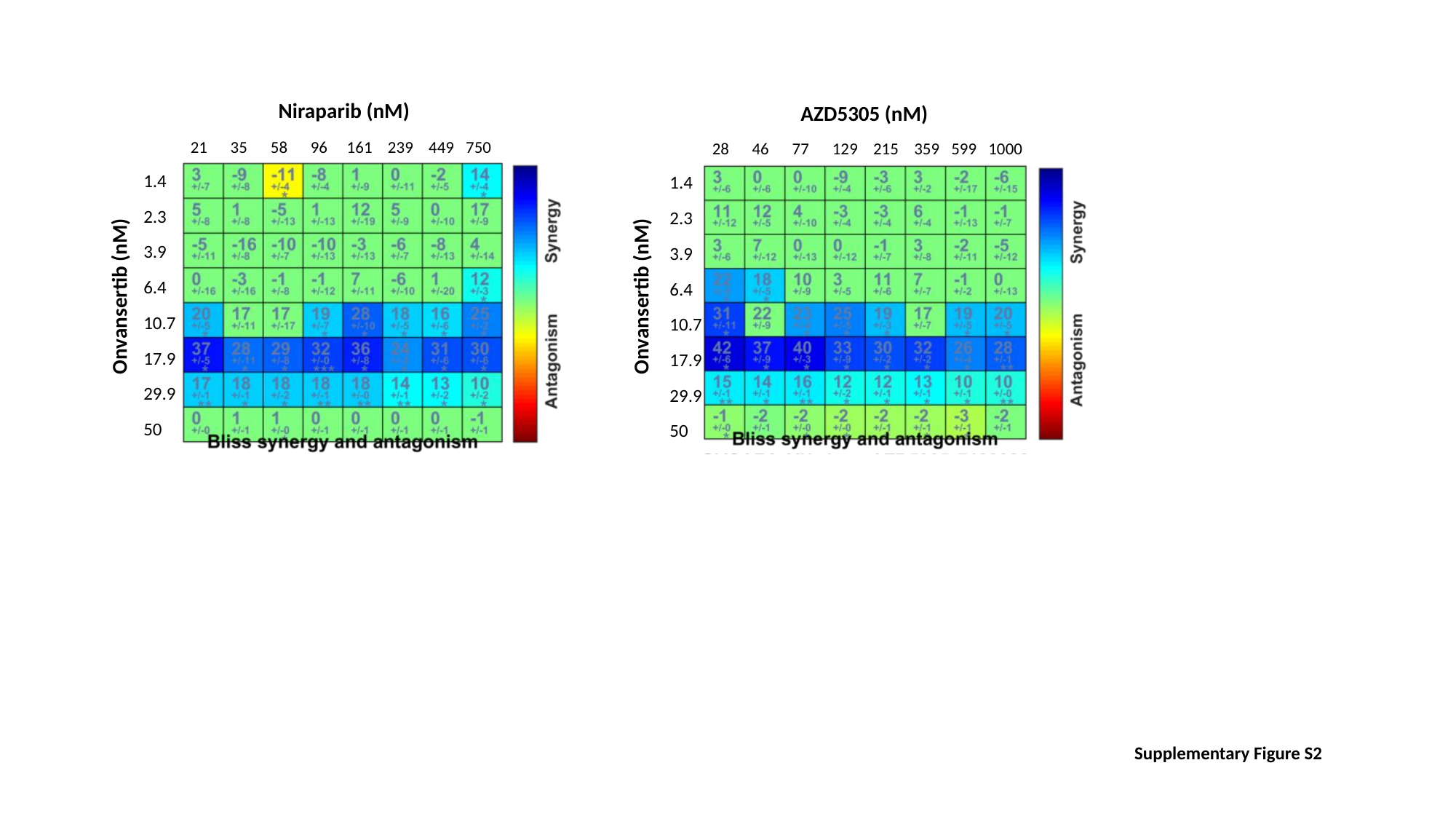

Niraparib (nM)
AZD5305 (nM)
21 35 58 96 161 239 449 750
28 46 77 129 215 359 599 1000
1.4
2.3
3.9
6.4
10.7
17.9
29.9
50
1.4
2.3
3.9
6.4
10.7
17.9
29.9
50
Onvansertib (nM)
Onvansertib (nM)
Supplementary Figure S2

## Slide 3
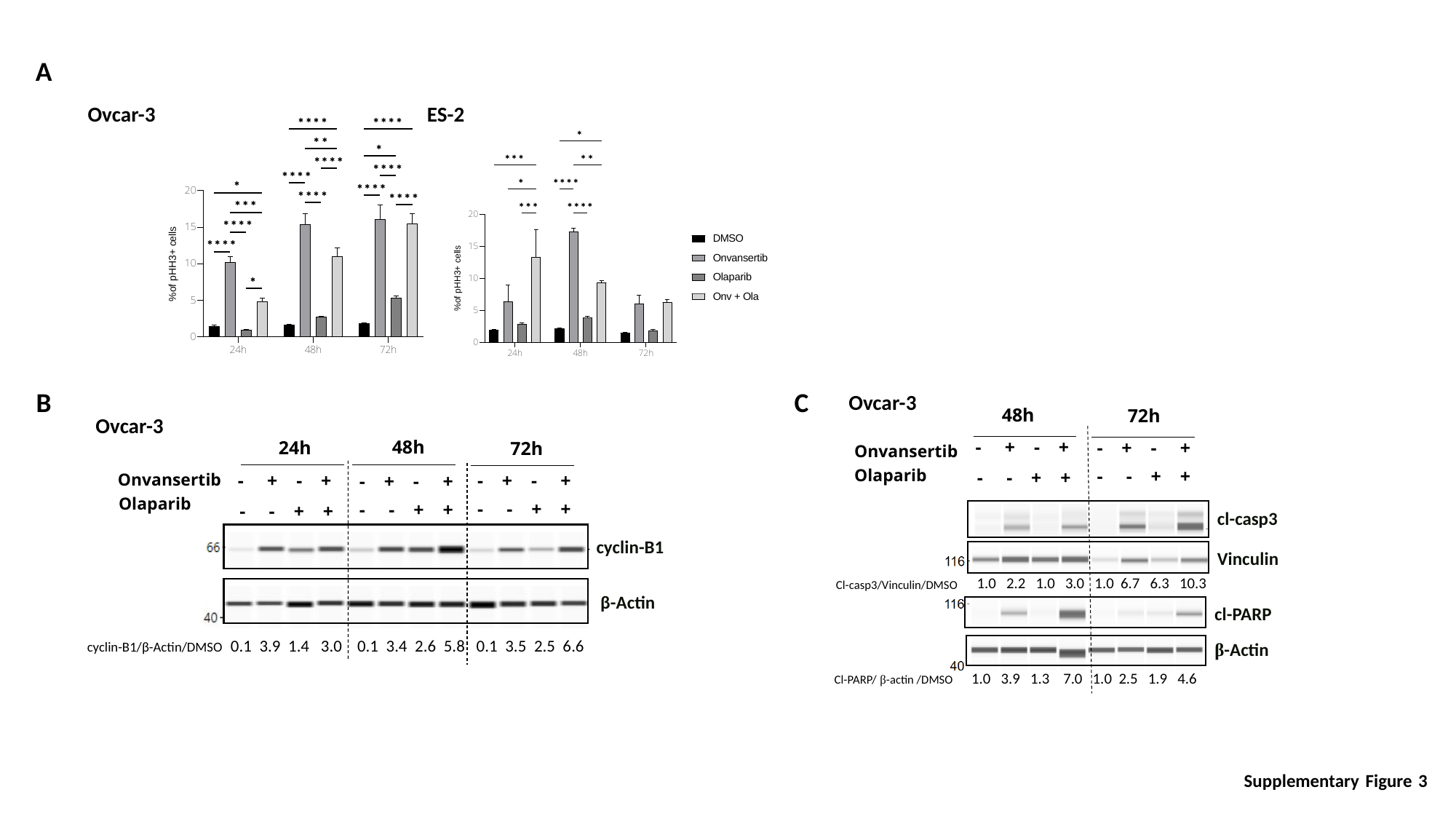

A
Ovcar-3
ES-2
B
Ovcar-3
48h
24h
72h
Onvansertib
Olaparib
cyclin-B1
β-Actin
 0.1 3.9 1.4 3.0 0.1 3.4 2.6 5.8 0.1 3.5 2.5 6.6
cyclin-B1/β-Actin/DMSO
C
Ovcar-3
48h
72h
- + - +
- + - +
Onvansertib
- - + +
Olaparib
- - + +
1.0 2.2 1.0 3.0 1.0 6.7 6.3 10.3
Cl-casp3/Vinculin/DMSO
cl-PARP
β-Actin
1.0 3.9 1.3 7.0 1.0 2.5 1.9 4.6
Cl-PARP/ β-actin /DMSO
cl-casp3
Vinculin
- + - +
- + - +
- + - +
- - + +
- - + +
- - + +
Supplementary Figure 3

## Slide 4
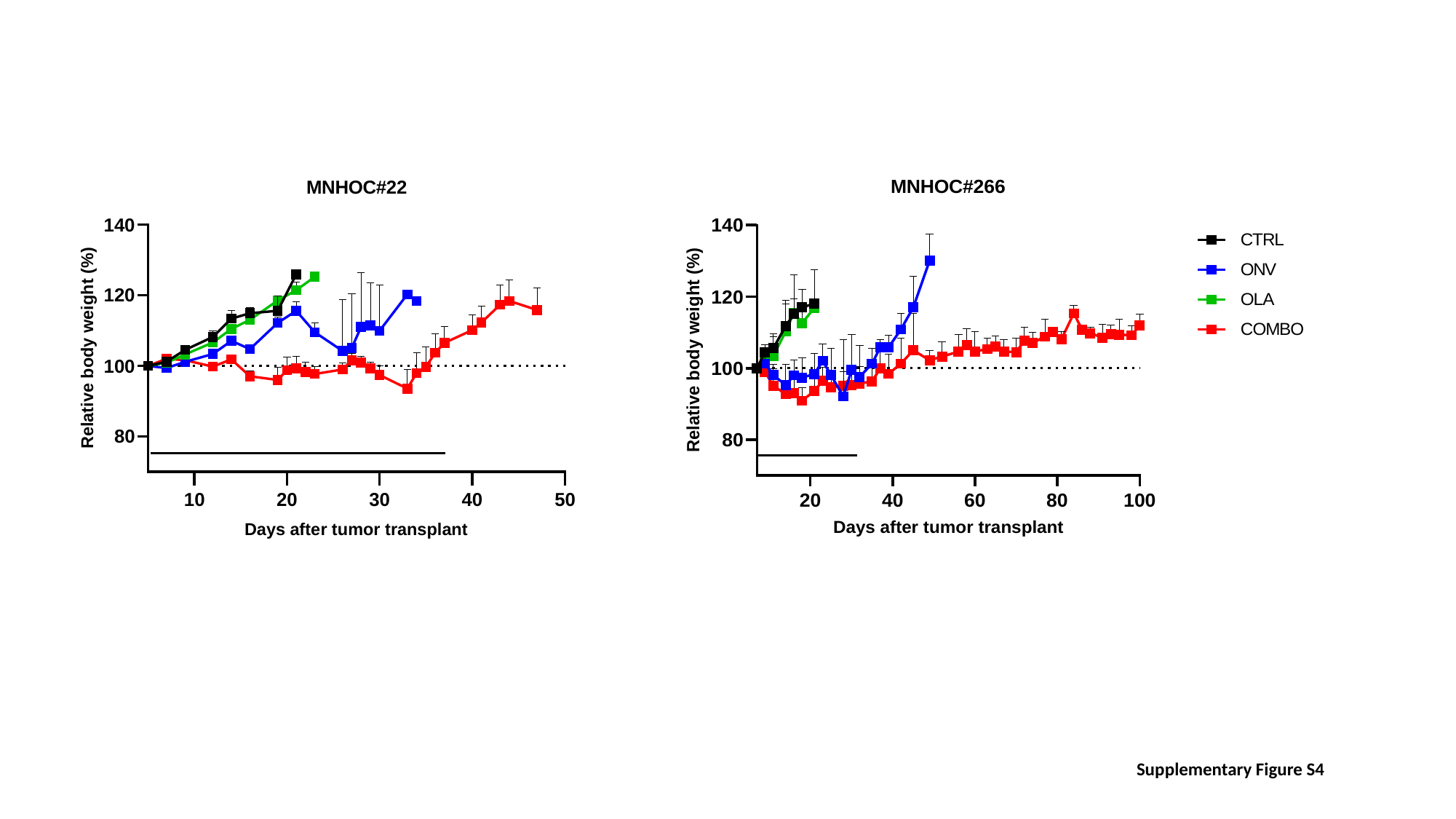

Supplementary Figure S4

## Slide 5
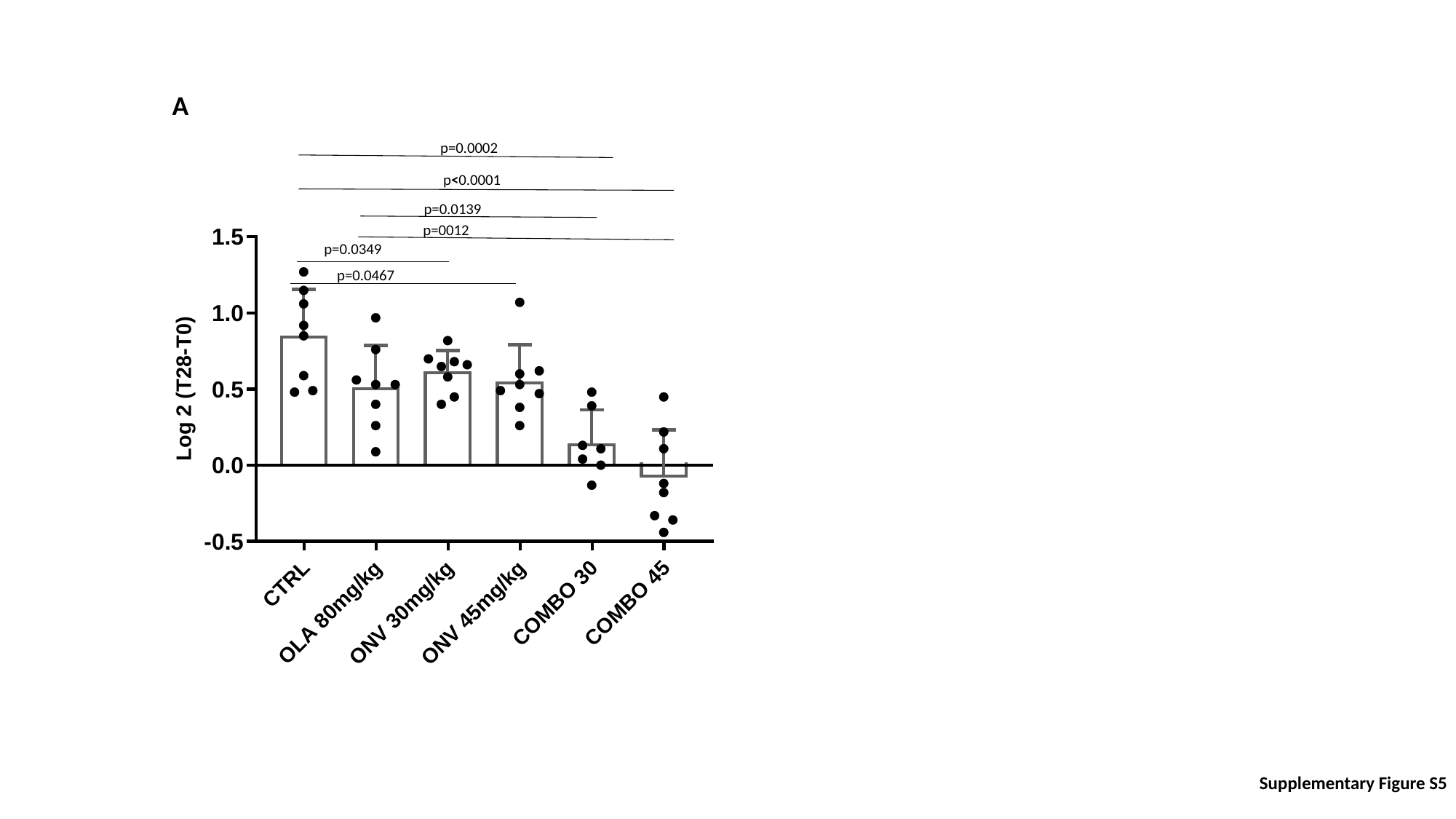

A
p=0.0002
p<0.0001
p=0.0139
p=0012
p=0.0349
p=0.0467
Supplementary Figure S5

## Slide 6
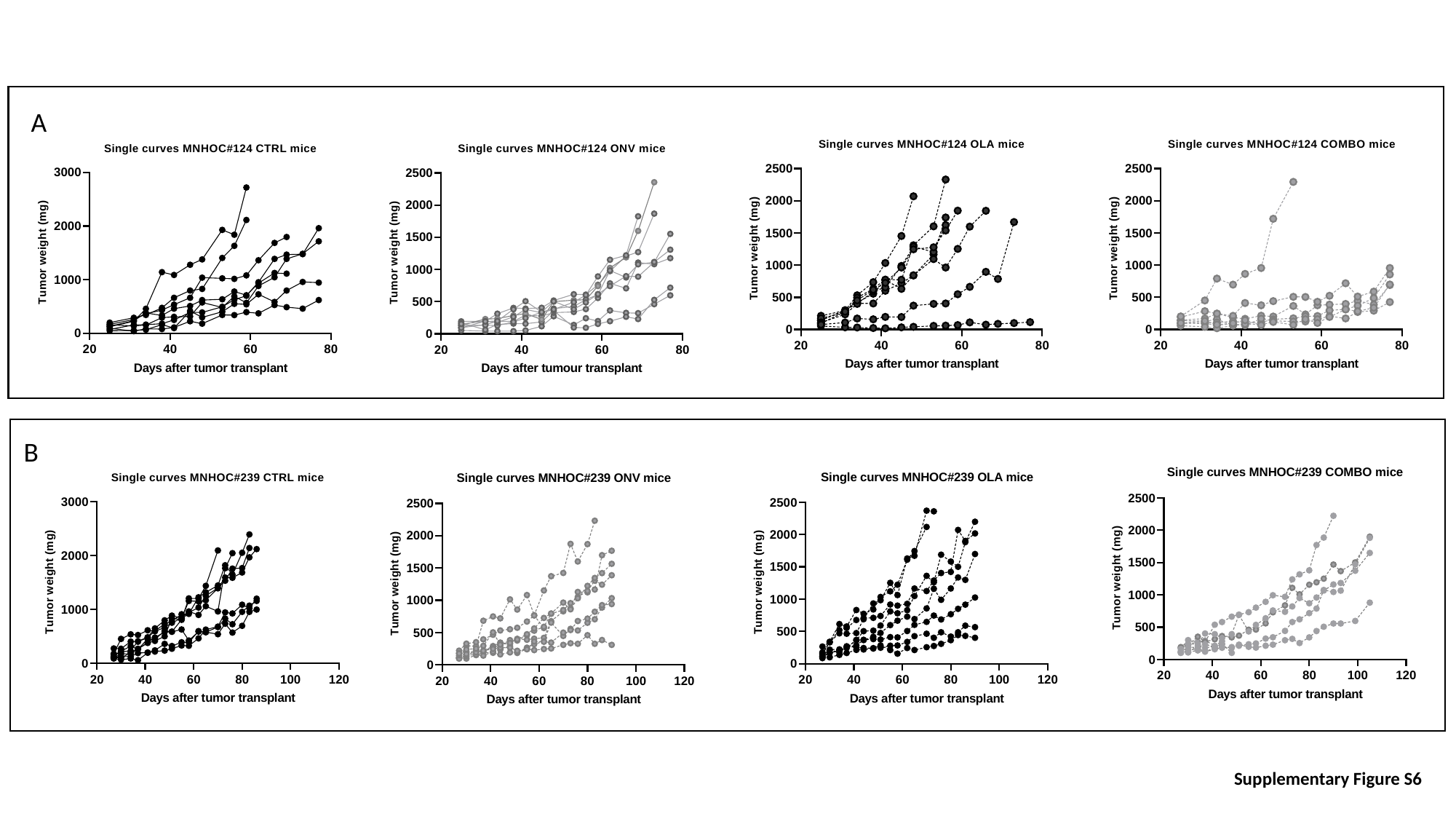

A
B
Supplementary Figure S6

## Slide 7
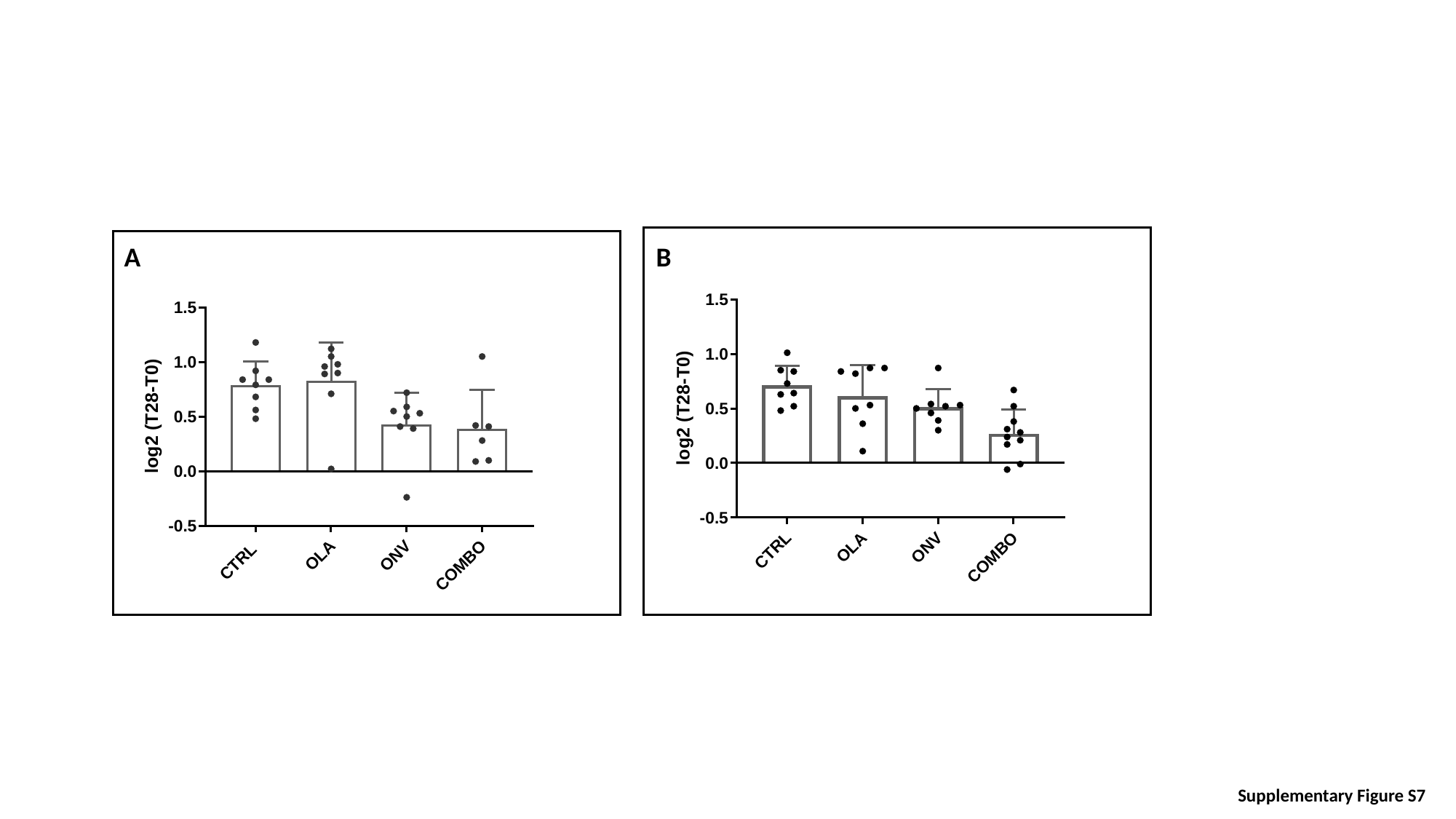

A
B
Supplementary Figure S7

## Slide 8
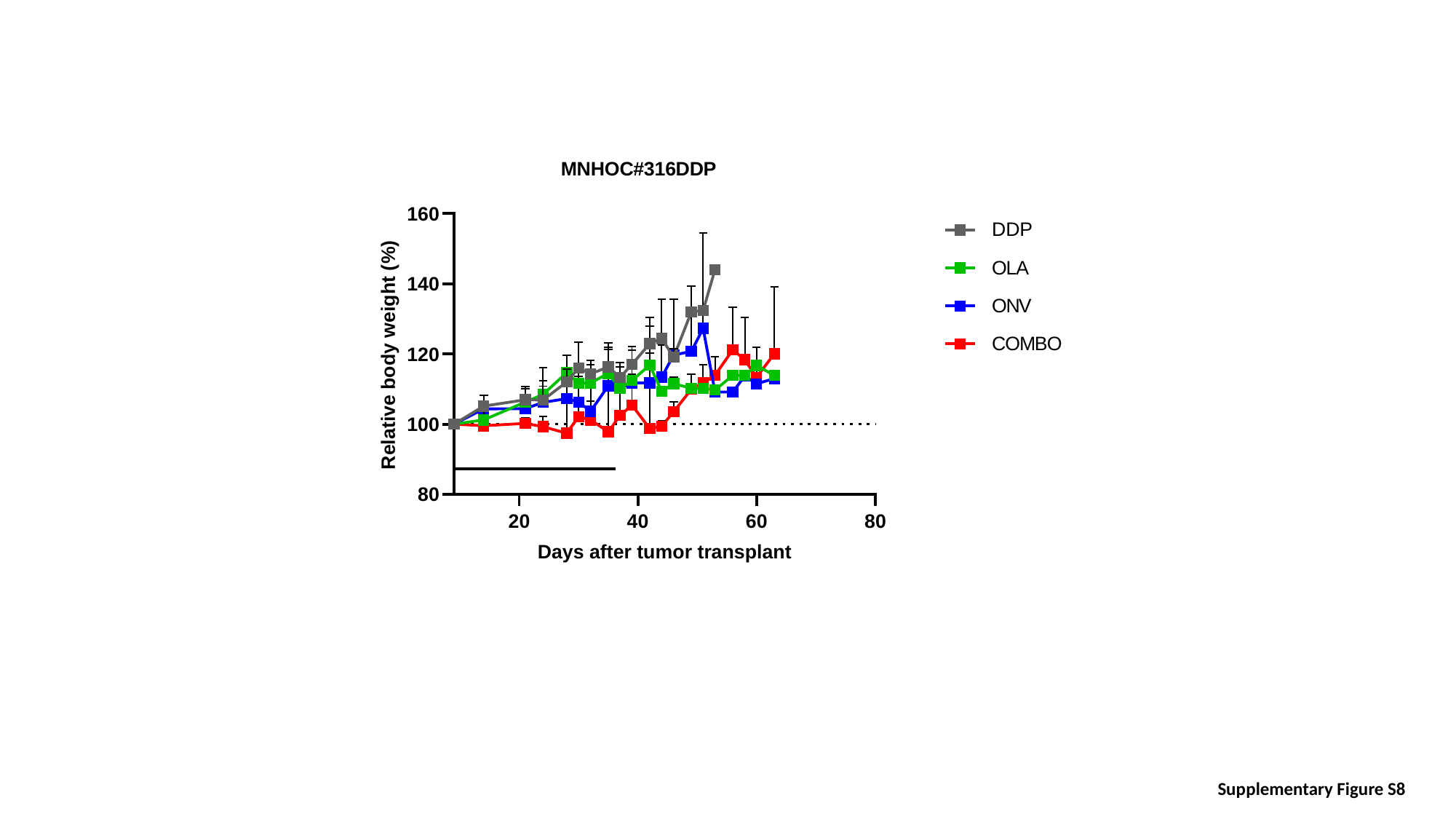

Supplementary Figure S8

## Slide 9
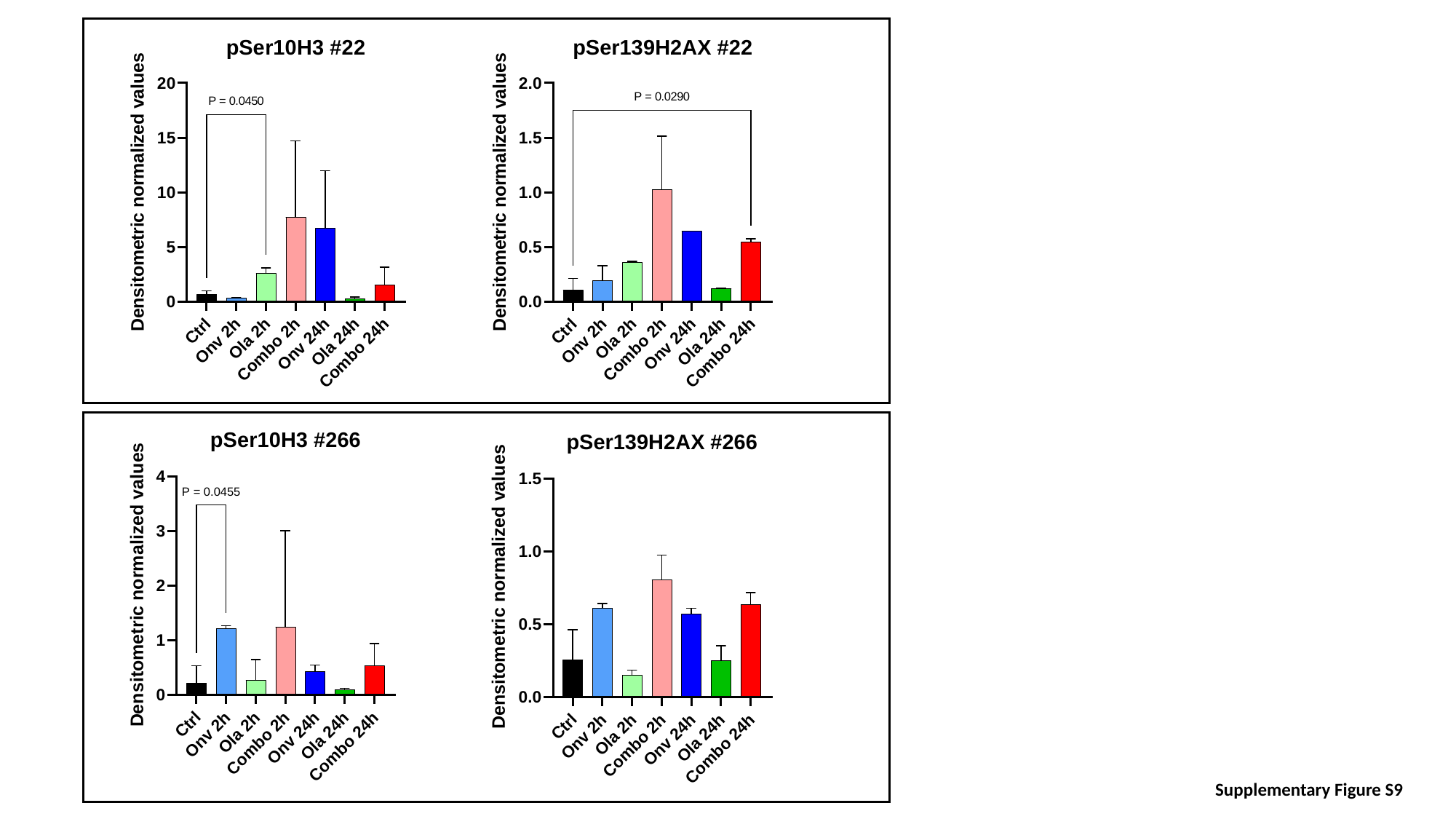

Supplementary Figure S9

## Slide 10
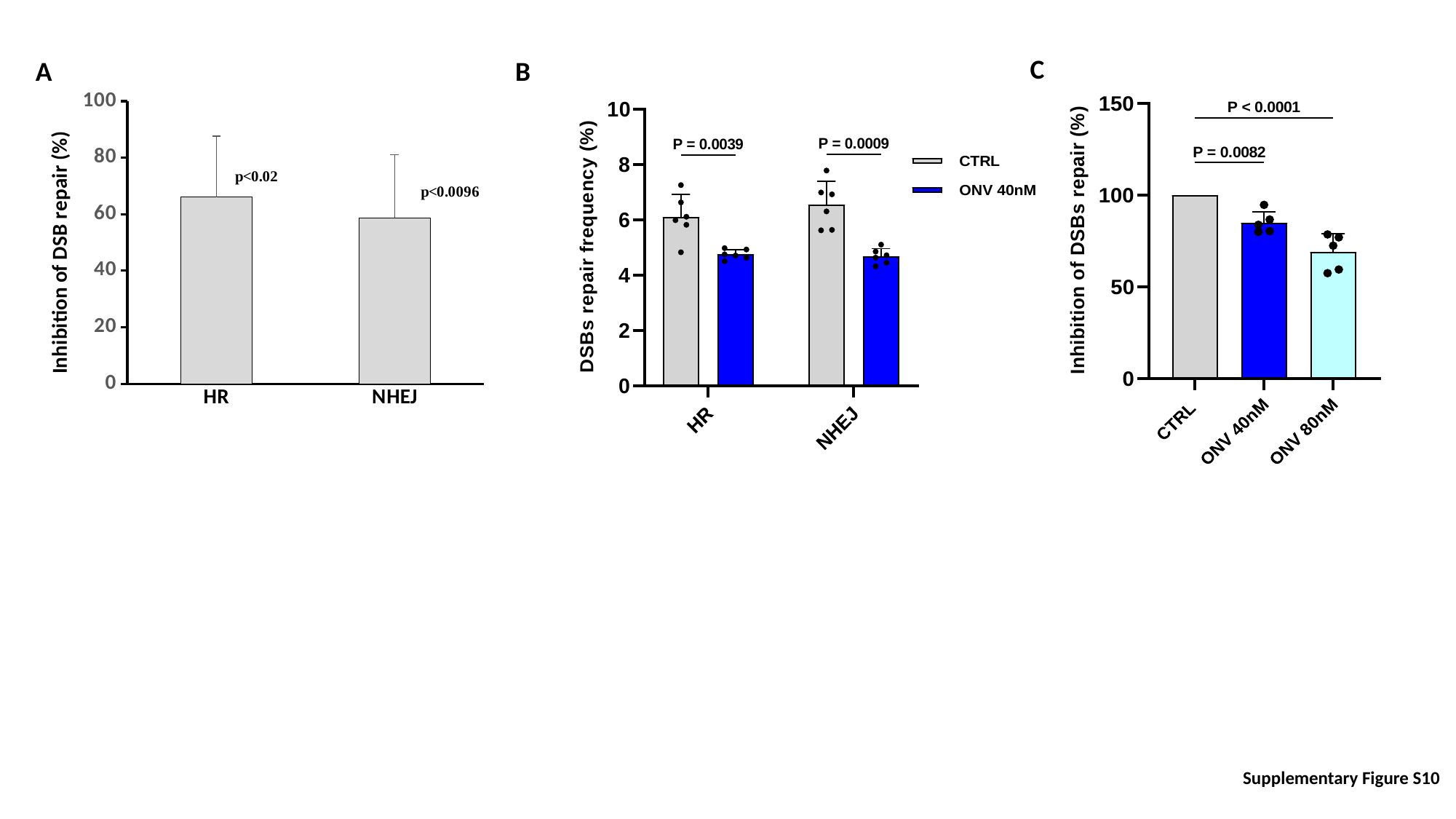

C
A
B
### Chart
| Category | |
|---|---|
| HR | 66.10514342410602 |
| NHEJ | 58.72846311786143 |
Supplementary Figure S10

## Slide 11
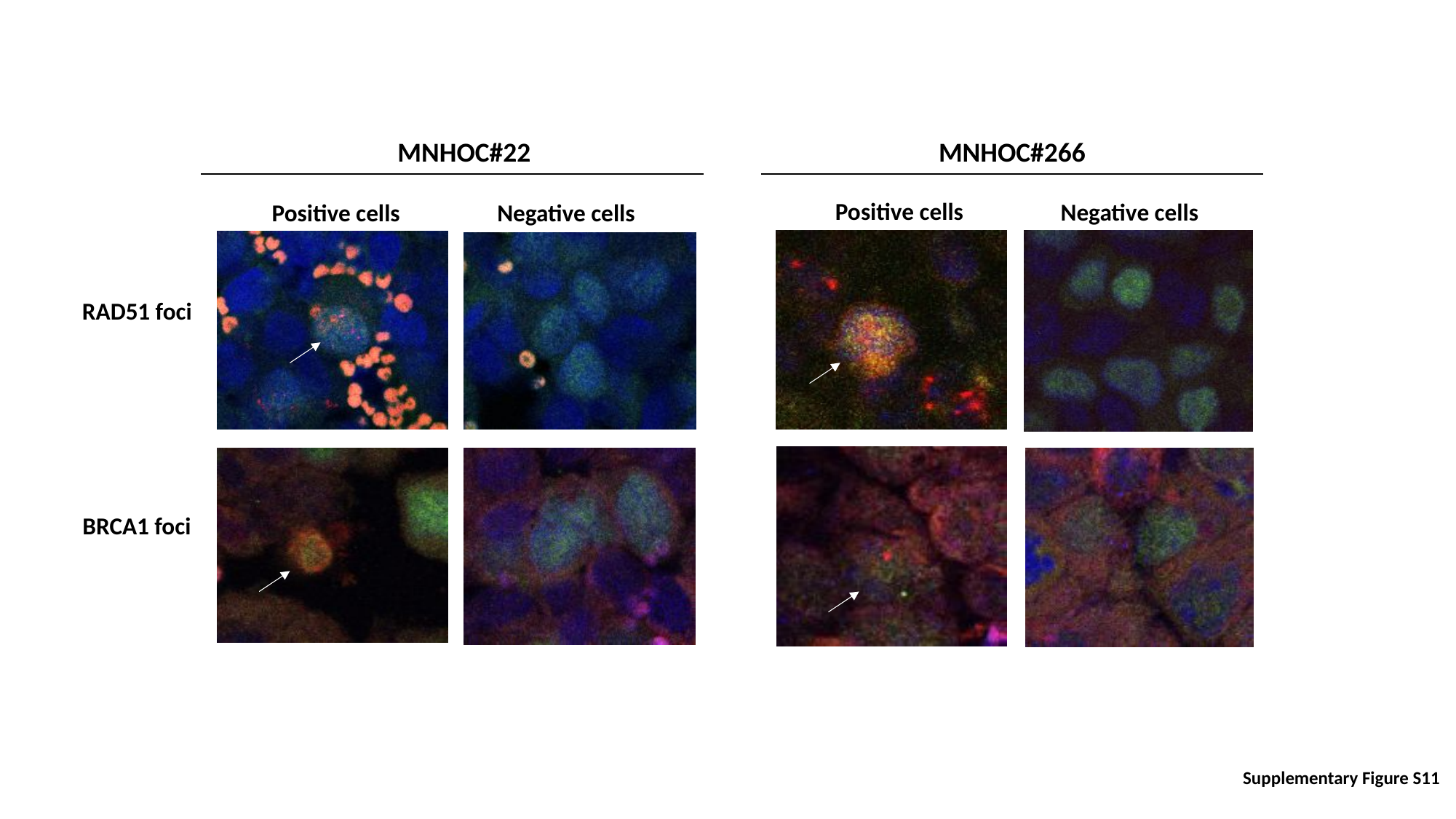

MNHOC#22
Positive cells
Negative cells
MNHOC#266
Positive cells
Negative cells
RAD51 foci
BRCA1 foci
Supplementary Figure S11

## Slide 12
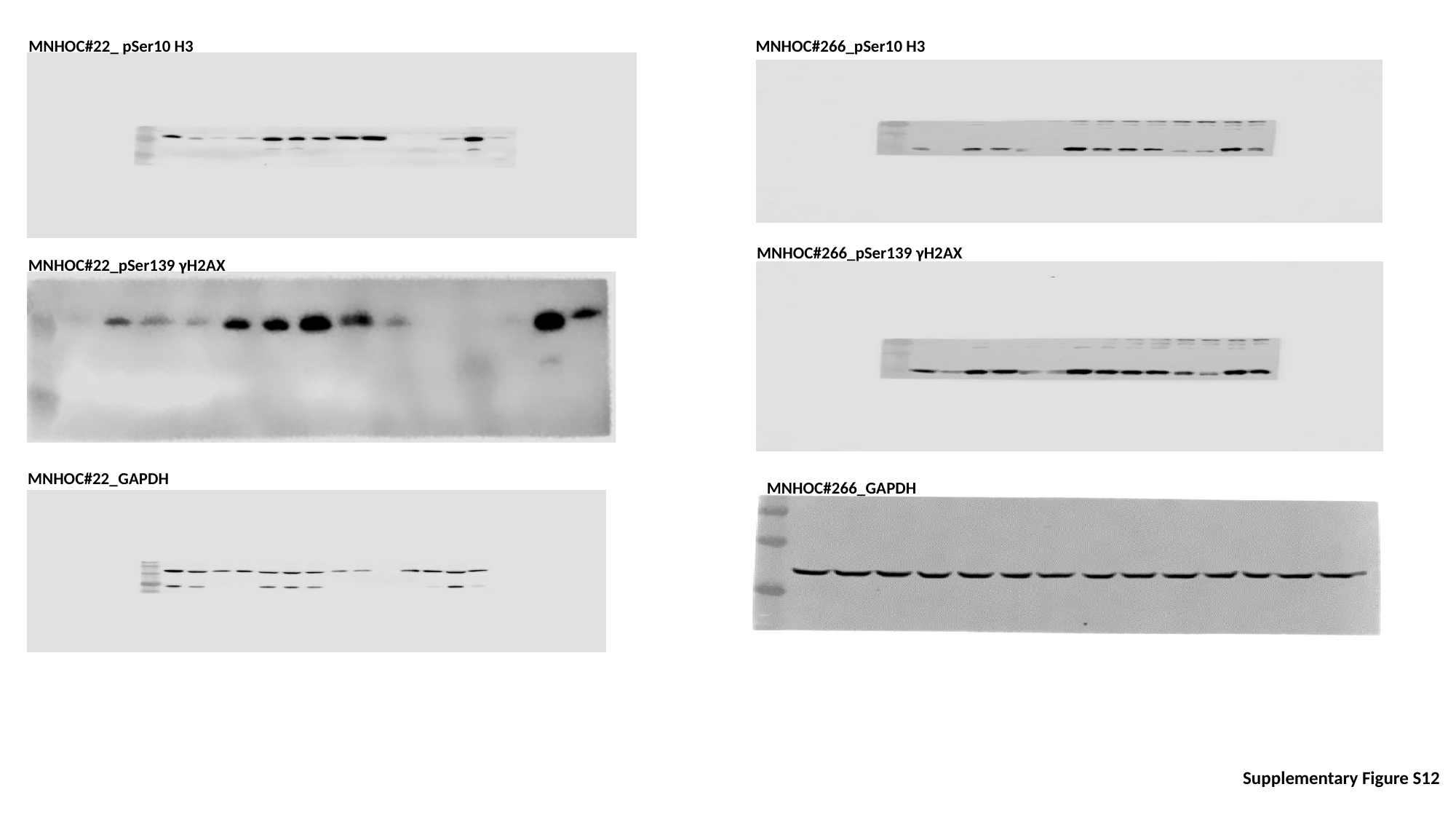

MNHOC#22_ pSer10 H3
MNHOC#266_pSer10 H3
MNHOC#266_pSer139 γH2AX
MNHOC#22_pSer139 γH2AX
MNHOC#22_GAPDH
MNHOC#266_GAPDH
Supplementary Figure S12
