# Supplementary material for: Onvansertib treatment overcomes olaparib resistance in high-grade ovarian carcinomas
Source: Cell Death Dis. 2024 Jul 22;15(7):521. doi: 10.1038/s41419-024-06894-1 (PMC11263393; doi:10.1038/s41419-024-06894-1)
Supplement: Supplementary file 3 — Supplementary Figure 12 [file 41419_2024_6894_MOESM3_ESM.pptx]

## Slide 1
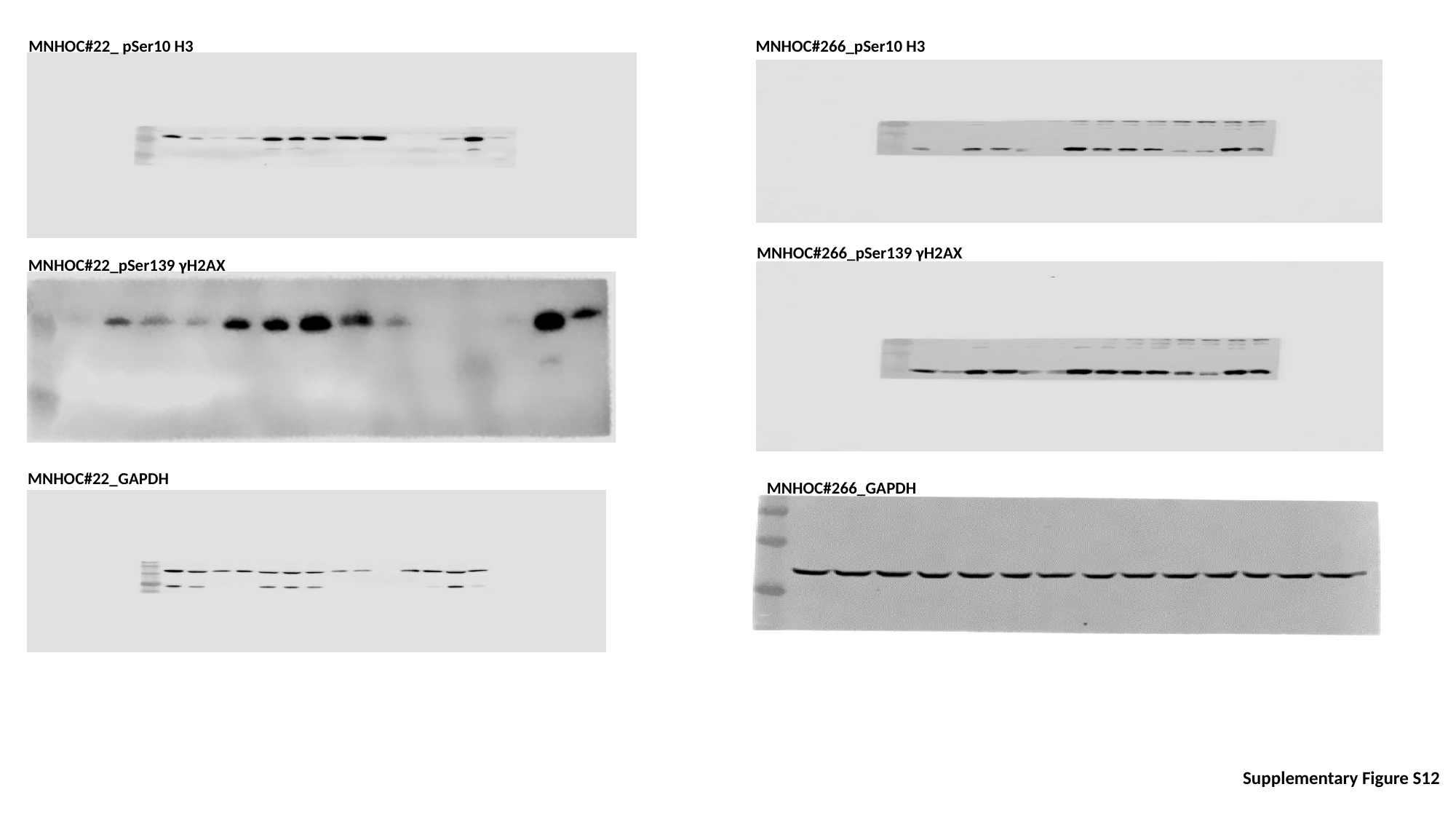

MNHOC#22_ pSer10 H3
MNHOC#266_pSer10 H3
MNHOC#266_pSer139 γH2AX
MNHOC#22_pSer139 γH2AX
MNHOC#22_GAPDH
MNHOC#266_GAPDH
Supplementary Figure S12
